# Structure, Regulation, and Inhibition of the Quorum-Sensing Signal Integrator LuxO

**DOI:** 10.1371/journal.pbio.1002464

**Published:** 2016-05-24

**Authors:** Hande Boyaci, Tayyab Shah, Amanda Hurley, Bashkim Kokona, Zhijie Li, Christian Ventocilla, Philip D. Jeffrey, Martin F. Semmelhack, Robert Fairman, Bonnie L. Bassler, Frederick M. Hughson

**Affiliations:** 1 Department of Molecular Biology, Princeton University, Princeton, New Jersey, United States of America; 2 Department of Biology, Haverford College, Haverford, Pennsylvania, United States of America; 3 Department of Chemistry, Princeton University, Princeton, New Jersey, United States of America; 4 Howard Hughes Medical Institute, Chevy Chase, Maryland, United States of America; Rutgers University-Robert Wood Johnson Medical School, UNITED STATES

## Abstract

In a process called quorum sensing, bacteria communicate with chemical signal molecules called autoinducers to control collective behaviors. In pathogenic vibrios, including *Vibrio cholerae*, the accumulation of autoinducers triggers repression of genes responsible for virulence factor production and biofilm formation. The vibrio autoinducer molecules bind to transmembrane receptors of the two-component histidine sensor kinase family. Autoinducer binding inactivates the receptors’ kinase activities, leading to dephosphorylation and inhibition of the downstream response regulator LuxO. Here, we report the X-ray structure of LuxO in its unphosphorylated, autoinhibited state. Our structure reveals that LuxO, a bacterial enhancer-binding protein of the AAA+ ATPase superfamily, is inhibited by an unprecedented mechanism in which a linker that connects the catalytic and regulatory receiver domains occupies the ATPase active site. The conformational change that accompanies receiver domain phosphorylation likely disrupts this interaction, providing a mechanistic rationale for LuxO activation. We also determined the crystal structure of the LuxO catalytic domain bound to a broad-spectrum inhibitor. The inhibitor binds in the ATPase active site and recapitulates elements of the natural regulatory mechanism. Remarkably, a single inhibitor molecule may be capable of inhibiting an entire LuxO oligomer.

## Abstract 2

Structural studies of the bacterial AAA+ enhancer-binding protein LuxO reveal the unprecedented mechanism of action of a small molecule quorum-sensing modulator that inhibits virulence in *Vibrio cholerae*.

## Author Summary

Bacteria communicate with one another and orchestrate group behaviors using a process called quorum sensing. The types of behaviors controlled by quorum sensing are typically unproductive when performed by a single bacterium acting alone but become effective when undertaken in unison by the group. Importantly, quorum sensing controls virulence and biofilm formation in many globally important pathogens. Thus, small molecules that modulate quorum sensing could be promising new antimicrobials. Here, we use X-ray crystallography to study LuxO, the central quorum sensing regulator in *Vibrio cholerae* and related bacteria. We find that LuxO activity is controlled during quorum sensing by a mechanism that entails occlusion of the LuxO active site by a linker region within the protein. The small molecule AzaU also inhibits LuxO and, in so doing, down-regulates *V*. *cholerae* virulence. Our studies show that AzaU binds to the LuxO active site, unexpectedly mimicking aspects of the natural regulatory mechanism.

## Introduction

**Fig 1:**
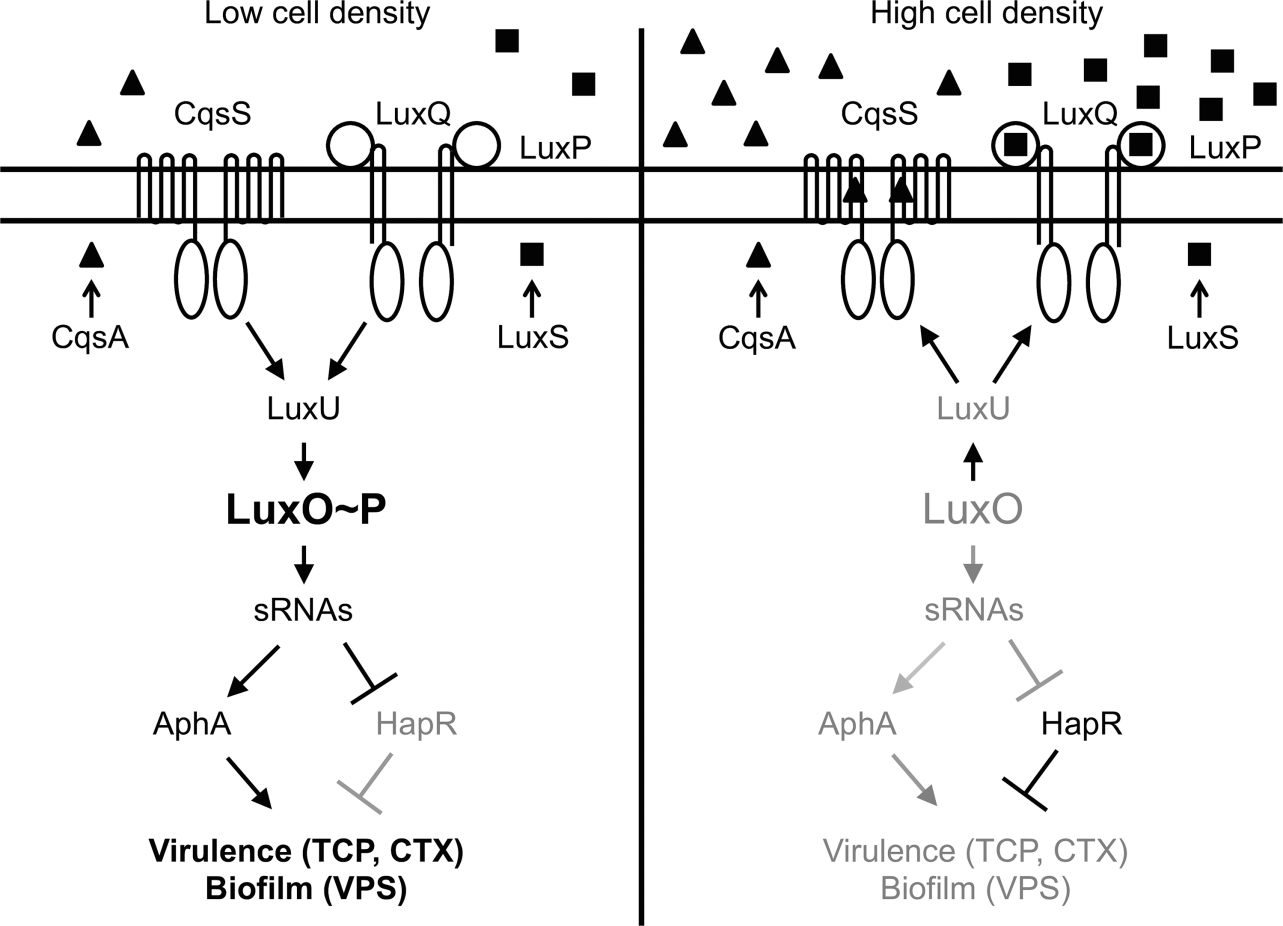
Quorum sensing in *V*. *cholerae*. Shown here are quorum-sensing circuits controlled by the autoinducers CAI-1 (▲) and AI-2 (■). At low cell density (left), autoinducer concentrations are below the detection threshold for their respective receptors, CqsS and LuxPQ. In the absence of bound autoinducer, these receptors funnel phosphate to a conserved aspartate (Asp 61) within the receiver domain of LuxO. Additional histidine sensor kinases, including CqsR and VpsS (not depicted), also shuttle phosphoryl groups to LuxO [2]. Phosphorylated LuxO activates the transcription of the genes encoding the small RNAs (sRNAs) Qrr1-4, which in turn post-transcriptionally activate AphA and repress HapR. At high cell density (right), binding of autoinducers to the receptors reverses the phosphorylation flow. LuxO is dephosphorylated, *qrr*1-4 are not transcribed, and HapR and AphA production are derepressed and inhibited, respectively. Genes, including those required for biofilm formation and virulence, are controlled by AphA and HapR.

Quorum sensing is a widespread process of bacterial cell–cell communication that allows bacteria to monitor and respond to fluctuations in cell number and the species composition of bacterial consortia. Quorum sensing relies on the production, release, and subsequent group-wide detection of extracellular signal molecules called autoinducers [1]. *Vibrio cholerae*, the etiological agent of the disease cholera, possesses multiple quorum-sensing pathways that function in parallel to regulate virulence factor production, biofilm formation, type VI secretion, and competence development, among other behaviors (Fig 1) [2–5]. Modulating quorum sensing might therefore be a strategy for mitigating pathogenicity [3,6–8].

The *V*. *cholerae* quorum-sensing receptors are membrane-bound two-component signal transduction proteins [2,3,7]. While each receptor detects a different autoinducer ligand, they all transduce autoinducer binding information to a shared response regulator called LuxO (Fig 1). At low cell density, when ligand is absent, the autoinducer receptors function as kinases and funnel ATP-derived phosphoryl groups to LuxO. Phosphorylated LuxO activates the transcription of genes encoding four small regulatory RNAs, Qrr1-4, which, in turn, control the translation of two key regulatory proteins, AphA and HapR (Fig 1) [9,10]. As a result, both virulence factor production and biofilm formation are activated. At high cell density, the binding of autoinducers to their cognate receptors inhibits receptor kinase activity, leading to the dephosphorylation and inactivation of LuxO. The resulting changes in AphA and HapR levels lead to the down-regulation of virulence factor production and biofilm formation. This counterintuitive pattern of behavior, in which virulence factor production and biofilm formation are inhibited at high cell density, can be understood in terms of the cholera disease itself [11]. Following successful infection, the ensuing diarrhea washes huge numbers of *V*. *cholerae* from the human intestine into the environment. Thus, expression of genes for virulence and biofilm formation at low cell density promotes infection, while repression of these genes by quorum-sensing autoinducers at high cell density promotes dissemination [3,12].

The central position of LuxO as the signal integrator in the quorum-sensing cascade controlling *V*. *cholerae* pathogenicity makes it an especially promising target for drug discovery. Furthermore, unlike other components of the quorum-sensing circuitry, LuxO is highly conserved in all sequenced vibrio species, including *V*. *parahaemolyticus* and *V*. *vulnificus*, which, like *V*. *cholerae*, are human pathogens [13]. By contrast, LuxO appears to be absent from the genomes of nonvibrio bacteria. Recently, we used high-throughput screening to identify small molecules that activate quorum sensing, and thus repress virulence factor production, in *V*. *cholerae* [8]. At least one of the compounds inhibited virulence by acting on LuxO. A more potent derivative, previously called compound 12 and here renamed AzaU, was shown to inhibit virulence factor production in both *V*. *cholerae* and *V*. *parahaemolyticus* [8].

LuxO belongs to the subfamily of AAA+ ATPases known as bacterial enhancer-binding proteins (bEBPs) [14,15]. Broadly speaking, AAA+ proteins exploit ATP hydrolysis to power mechanical work in processes such as protein unfolding, DNA unwinding, and transcriptional regulation [16]. bEBPs such as LuxO drive the “opening” of σ^54^-dependent promoters, converting them to transcriptionally activate states [14,15]. They do so by binding to enhancer-like sequences upstream of target promoters and interacting directly with the σ^54^ subunit of the RNA polymerase holoenzyme. bEBPs are ring-shaped hexamers, or possibly heptamers, in their active states [17]. Within the group I bEBPs, which includes LuxO and the well-studied NtrC proteins, each monomer contains three domains: an N-terminal receiver (R) domain, a central ATPase (C) domain, and a C-terminal sequence-specific DNA-binding (D) domain [14,15]. In LuxO, as in NtrC proteins, the phosphorylation of an aspartate located within the R-domain activates the C domain for ATP hydrolysis and the opening/activation of σ^54^-dependent promoters [18,19]. The DNA-binding domains direct LuxO transcriptional activation to the *qrr*1-4 genes [9].

Group I bEBPs can exhibit either negative or positive phosphorylation-dependent regulation [20–24]. In the negative mode of regulation, the R domain blocks the formation of active bEBP oligomers by stabilizing inactive dimers; these dimers are disrupted by R domain phosphorylation, permitting spontaneous ring assembly driven by the C domains [20,22,23]. In the positive mode of regulation, phosphorylated R domains are needed to drive oligomerization [21]. Here, we present crystal structures and in vitro and in vivo functional studies of LuxO that together reveal what is, to our knowledge, an entirely novel mechanism of AAA+ protein regulation, in which a regulatory segment of the protein prevents substrate ATP binding and hydrolysis by occupying a portion of the enzyme active site. Notably, this intrinsic regulatory mechanism is recapitulated by the small molecule inhibitor AzaU. Finally, our data suggest that a single AzaU inhibitor molecule is capable of inactivating an entire ring, placing constraints on potential mechanisms of AAA+ function.

## Results

### LuxO Crystal Structures

**Fig 2:**
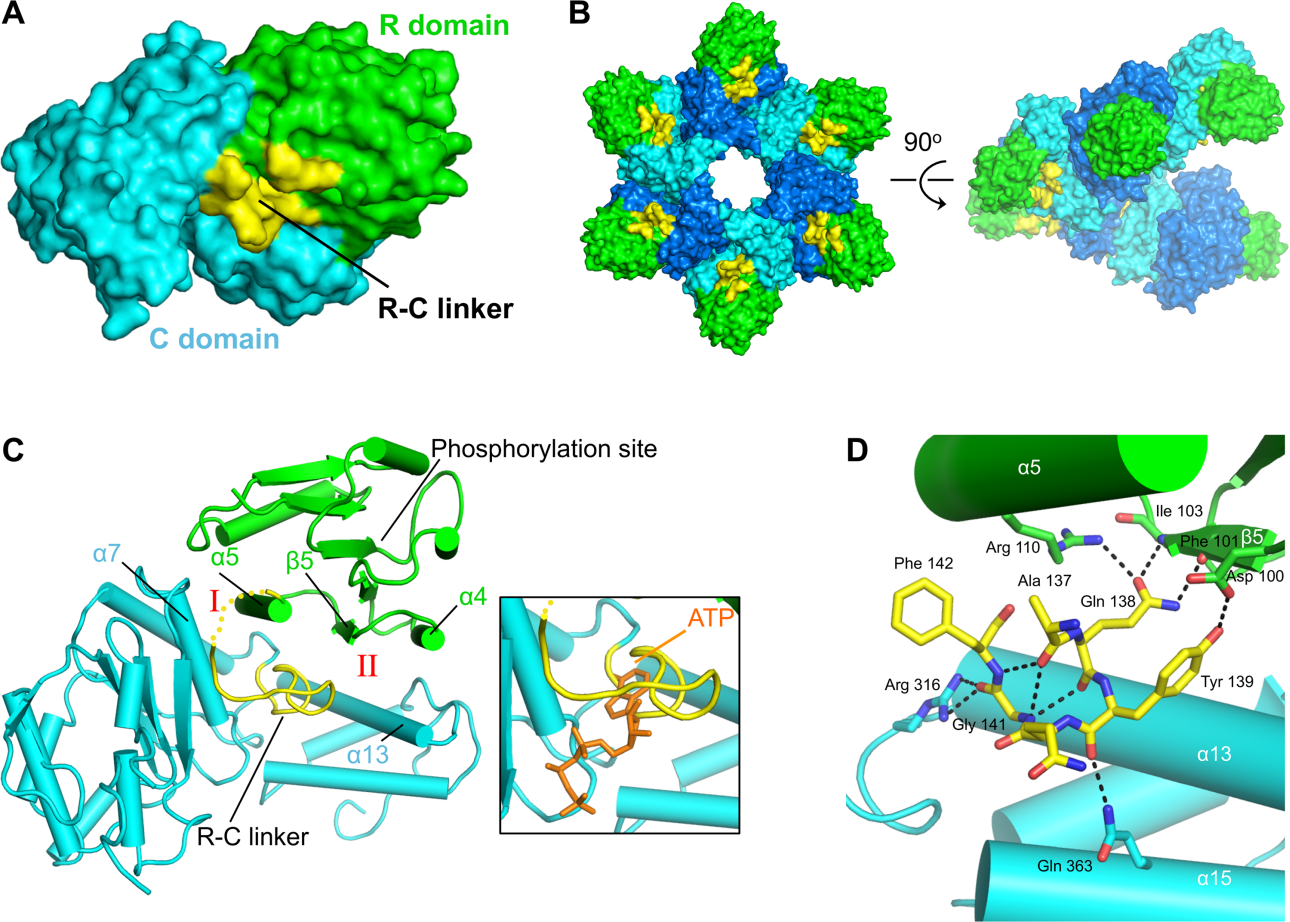
*V*. *angustum* LuxO-RC structure. (A) Each LuxO-RC monomer contains a receiver (R) domain (green), a linker (R-C linker, yellow), and a catalytic central (C) domain (cyan). Because of crystal symmetry, all LuxO-RC monomers are identical. (B) Within the crystals (space group P6_1_), the LuxO-RC monomers form continuous helices with a 6-fold repeat. The intermonomer contacts closely resemble those observed in other AAA+ ATPases, such as NtrC1 [22], that crystallize in flat six- or seven-membered rings. Coloring is as in panel A, except that, for clarity, every other C domain is colored blue. (C) The LuxO-RC monomer, colored as in panel A. The molecule is rotated, relative to panel A, to provide a clear view of the R domain-C domain interface and the R-C linker. Interfaces I and II, described in the text, are indicated. In the inset, ATP (modeled based on the LuxO-C:ATP structure) is included to illustrate that the R-C linker occupies a substantial portion of the ATPase active site. (D) An H-bonding network involving both R and C domain residues stabilizes the R-C linker in the LuxO active site.

Previously, we reported that *V*. *harveyi* LuxO lacking the R domain is constitutively active in vivo [18]. This result implies that LuxO is negatively regulated by its R domain and that R domain phosphorylation releases this negative regulation. To investigate the mechanism of intrinsic LuxO regulation further, we used X-ray crystallography. Of the seven vibrio LuxO proteins we tested, *V*. *angustum* LuxO proved most amenable to structural studies (S1 Fig; S1–S3 Tables). We determined the 1.6 Å resolution crystal structure of a *V*. *angustum* LuxO construct lacking the D domain but containing both R and C domains (denoted LuxO-RC) (Fig 2A, S2 Table). The structures of the individual R and C domain are very similar to those of homologs such as NtrC1 (root mean square [rms] deviations of 1.3 Å and 1.7 Å, respectively), although, as discussed below, the relative positioning of the R and C domains is unique. Rather than forming closed rings, *V*. *angustum* LuxO monomers in the crystals form continuous helical arrays with six subunits per turn (Fig 2B). We also observed the same helical arrays of LuxO monomers in crystal structures of the *V*. *angustum* C domain alone (LuxO-C), either as the apo-protein, with ATP bound, or with the inhibitor AzaU bound (S3 Table; discussed below). Both closed rings and helical arrays of various pitches are common among the known crystal structures of AAA+ ATPases. Presumably, because only a modest alteration in the interaction between neighboring monomers within a flat ring is required to generate a helix, the crystallographically observed arrangements often reflect those that are favored by symmetry considerations and crystal packing forces. Indeed, the monomer–monomer interfaces observed in our LuxO-RC and LuxO-C structures are similar to those observed in NtrC1 [22] and other AAA+ proteins that crystallize in closed-ring arrangements. Sedimentation velocity analytical ultracentrifugation experiments suggest that, in solution, LuxO-RC forms hexamers (discussed below; see Fig 5B).

In the *V*. *angustum* LuxO-RC structure, the R domain is nestled between the two lobes of the C domain, forming a discrete interface with each (Fig 2C). Interface I is dominated by an interaction between α-helix 5 (α5) of the R domain and α7 of the C domain. In interface II, α4 and β-strand 5 (β5) of the R domain interact with α13 of the C domain. The R and C domains are connected by a 20-residue linker (denoted R-C linker, residues 123–142). Strikingly, residues 137–142 of the R-C linker occupy a substantial portion of the C domain active site, sterically occluding the binding of the ATP substrate (Fig 2C). The R-C linker is stabilized in the active site by an extensive network of H-bonds, including four with residues within the R domain and three with residues within the C domain (Fig 2D). Gly 141 of the R-C linker appears to be especially important, as it not only makes three key H-bonds using its main chain nitrogen and oxygen atoms but also occupies a position deep within the active site that could not accommodate a larger residue. The positioning we observe for the R domain in relation to the C domain and the insertion of the R-C linker into the catalytic active site have not to our knowledge been observed for any other bEBP or AAA+ protein and thus appear to represent an unprecedented mode of AAA+ protein regulation.

### Functional Validation of the Implications of the LuxO Crystal Structure

**Fig 3:**
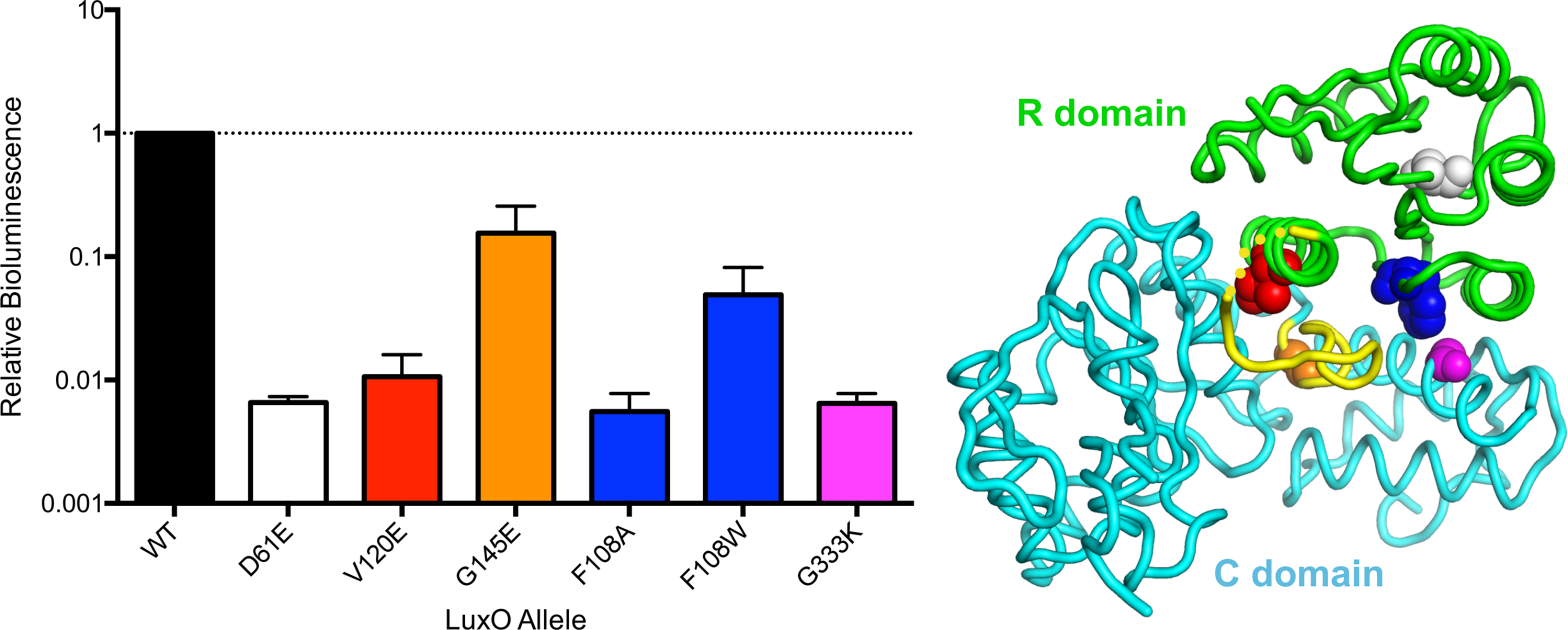
The autoinhibited structure of *V*. *angustum* LuxO-RC is physiologically relevant. Bioluminescence produced by a *V*. *cholerae* reporter strain carrying LuxO-controlled luciferase. Light production values for mutant strains are normalized to that produced by inactive, wild-type LuxO (WT). Mutations introduced into *V*. *cholerae* LuxO with the goal of disrupting the R-C interface display reduced bioluminescence—signifying increased LuxO activity—comparable to the constitutively active phosphomimetic LuxO D61E. The underlying data can be found in S1 Data.

To validate the physiological relevance of the domain and linker interactions that we observe in our structure with respect to the regulation of LuxO activity, we engineered mutations in the highly homologous (66% sequence identity, 78% sequence similarity) *V*. *cholerae* LuxO protein (S1 Fig, S4 Table). The mutations were designed to disrupt the R-C interface or, in one case, to change the key R-C linker glycine (Gly 141) and were thereby expected to derepress (i.e., activate) LuxO. LuxO activation is readily assayed using a *V*. *cholerae* reporter strain containing a quorum-sensing controlled luciferase operon [8]. This recombinant *V*. *cholerae* strain produces light exclusively when LuxO is inactive—that is, at high cell density in the presence of autoinducers (see Fig 1). Mutations that activate LuxO confer the low-cell-density phenotype and cause reduced bioluminescence. As a positive control for LuxO activation, we used an isogenic *V*. *cholerae* strain carrying the constitutively active phosphomimetic mutant LuxO D61E (earlier misnamed D47E), which reduces bioluminescence about 100-fold compared to the strain carrying wild-type LuxO (Fig 3).

Interface I was targeted by changing a central residue in the α5–α7 interaction, Val 120 (Ile 113 in *V*. *angustum* LuxO), to glutamate. This LuxO V120E mutant exhibited 100-fold reduced bioluminescence compared to wild-type LuxO (Fig 3), signifying strong LuxO activation upon interface I disruption. Interface II was targeted by changing the R domain residue Phe 108 (Phe 101 in *V*. *angustum* LuxO) to alanine (F108A) or tryptophan (F108W) or by changing the C domain residue Gly 333 (Gly 329 in *V*. *angustum* LuxO) to lysine (G333K). These mutants displayed 10-fold to 100-fold reduced bioluminescence (Fig 3). Taken together, these results strongly support the physiological relevance of the crystallographically defined R-C interfaces. Finally, we note that *V*. *cholerae* LuxO Gly 145 (Gly 141 in *V*. *angustum* LuxO), whose small size allows the R-C linker to fit into the ATP-binding site (Fig 2D), is conserved in all LuxO proteins but not in the otherwise closely related NtrC proteins (S1 Fig). Changing this LuxO residue to glutamate (G145E), the residue located at this position in NtrC1, reduced bioluminescence 10-fold (Fig 3), validating a direct role for the R-C linker in C domain inhibition. Nonetheless, this residual level of bioluminescence implies that LuxO inhibition has not been completely eliminated, despite the predicted inability of the linker to occupy the ATPase active site. The LuxO-RC crystal structure provides an attractive explanation, showing that the R domain interacts with both lobes of the C domain. These interactions may impede interlobe movements needed for the catalytic cycle.

**Fig 4:**
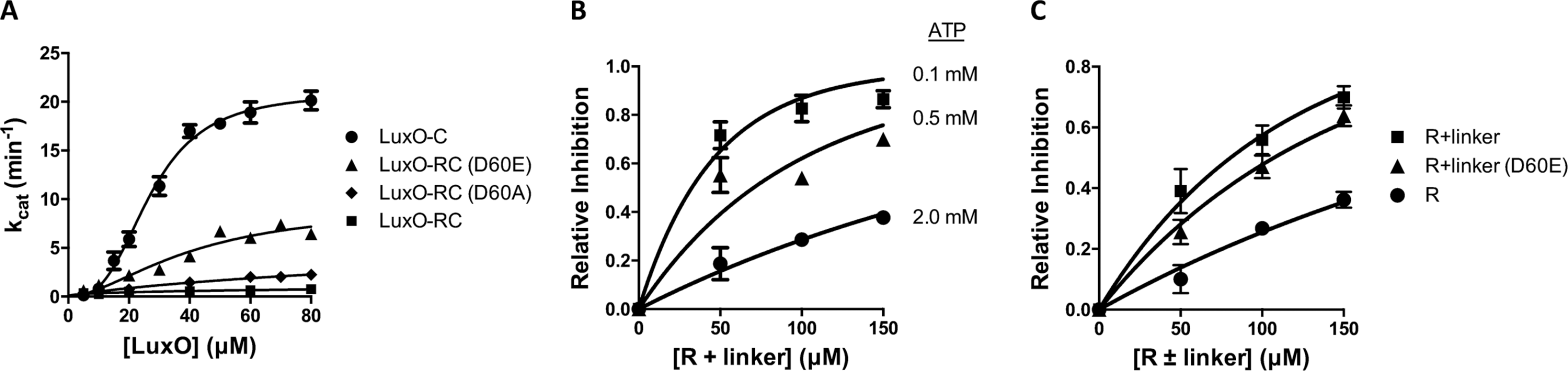
The R domain and R-C linker are negative regulators of *V*. *vulnificus* LuxO ATPase activity. (A) Enzyme turnover number was measured using a coupled ATPase assay. Shown are LuxO-C, LuxO-RC (wild-type), and LuxO-RC phosphomimetic (R60E) and control (R60A) mutants. (B) The ATPase activity of LuxO-C (100 μM) was measured in the presence of increasing concentrations of LuxO-R+linker (residues 1–146) at three different ATP concentrations. Data are plotted as inhibition relative to LuxO-C only. (C) The ATPase activity of LuxO-C (100 μM) was measured in the presence of increasing concentrations of LuxO-R+linker, LuxO-R+linker (D60E), and LuxO-R (residues 1–128). ATP concentration was 0.5 mM. In all panels, error bars represent standard deviation (*n* = 3); where no error bar is visible, it is smaller than the corresponding symbol. The data underlying panels A–C can be found in S1 Data.

**Fig 5:**
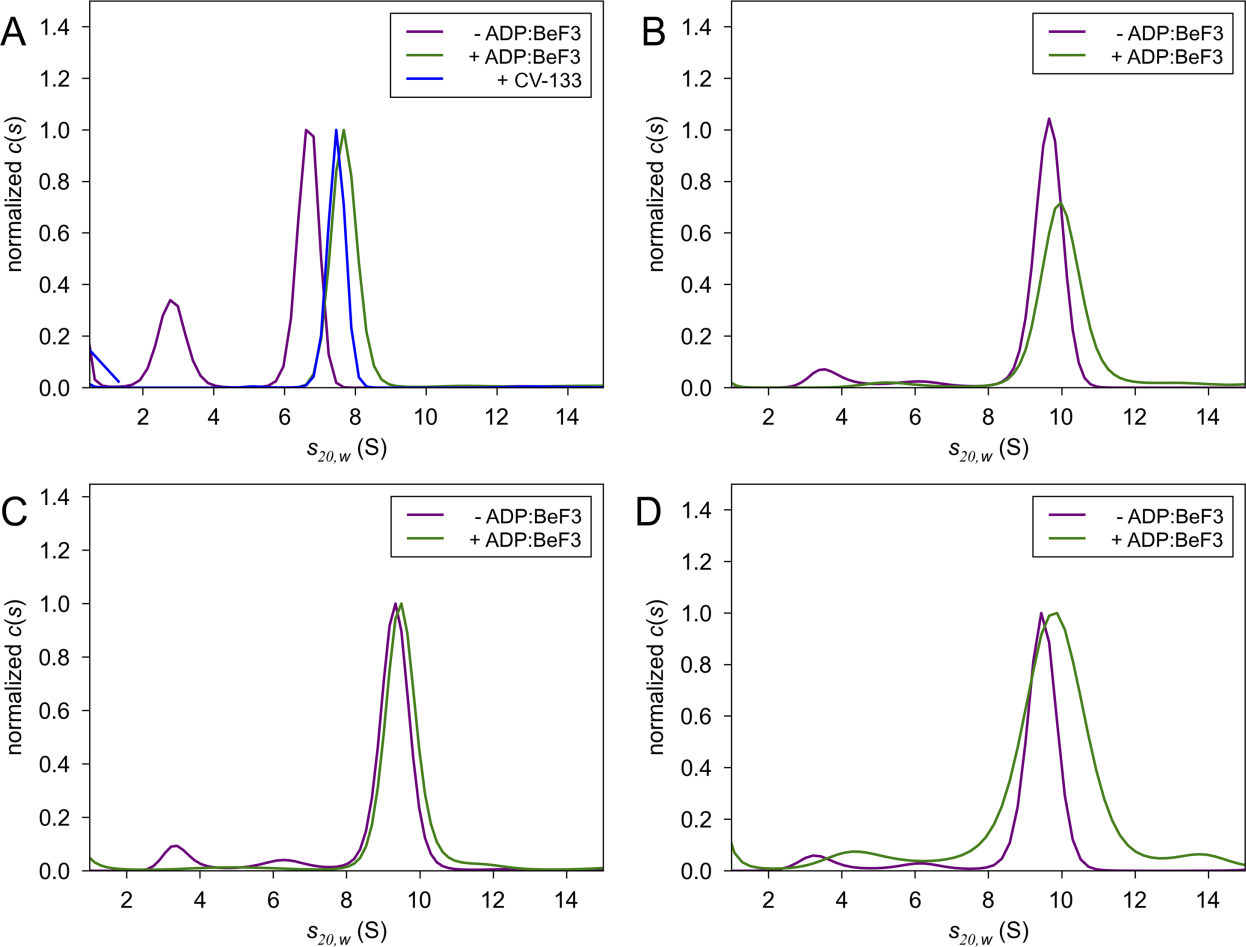
*V*. *vulnificus* LuxO-C and LuxO-RC are predominantly hexamers in solution. Sedimentation velocity analytical ultracentrifugation was used to evaluate the oligomerization states of (A) 100 μM LuxO-C, (B) 100 μM LuxO-RC (wild type), (C), LuxO-RC (D60A), and (D) LuxO-RC (D60E). In each panel, one sample included ADP:BeF_3_ as a substrate/transition state analog. Also shown in panel A is a sample containing 60 μM LuxO-C and 120 μM CV-133, a potent competitive inhibitor. While BeF_3_ could exert an activating effect on wild-type LuxO-RC by mimicking the phosphoryl group on Asp 60, it is highly unlikely to have this effect on the D60A or D60E mutant LuxO-RC proteins. See text and Materials and Methods for further details.

A prediction of our structure-based model for LuxO regulation is that, at sufficiently high concentrations, a construct containing the R domain and R-C linker might be capable of inhibiting the ATPase activity of the C domain in trans. To lay the groundwork for testing this possibility, we developed an in vitro LuxO ATPase activity assay. Of the vibrio species we evaluated, *V*. *vulnificus* LuxO-C (88%/82% sequence similarity/identity with *V*. *cholerae* LuxO; S1 Fig) displayed the most robust ATPase activity and was therefore selected for enzyme assays. As expected for an enzyme whose activity relies on oligomerization, the measured rate constant k_cat_ displayed a sigmoidal dependence on LuxO concentration (Fig 4A). Using sedimentation velocity analytical ultracentrifugation, we confirmed that, at the LuxO-C concentration required for full activity, the protein was almost exclusively hexameric (Fig 5A and Materials and Methods). We note, however, that the concentration of LuxO-C required for full activity was nonphysiologically high; this was also the case for the other vibrio LuxO C domains we tested (S1 Table). This result may reflect the absence of the DNA-binding domain; unfortunately, the propensity of full-length LuxO proteins to aggregate has, so far, prevented us from investigating this possibility more fully. Despite this complication we found that, consistent with the negative regulatory role of the R domain on the C domain, LuxO-RC was almost entirely inactive compared to LuxO-C (Fig 4A). Furthermore, the phosphomimetic LuxO-RC mutant D60E (equivalent to *V*. *cholerae* LuxO D61E), was substantially activated, whereas a control LuxO-RC D60A mutant that mimics the unphosphorylated form was, like LuxO-RC, inactive. Together, these experiments establish that purified LuxO-RC retains the key regulatory features identified in previous in vivo studies of LuxO.

With a LuxO ATPase assay in hand, we could test whether a construct containing the R domain and R-C linker (“LuxO-R+linker”) was capable of inhibiting the C domain in trans. Indeed, we observed dose-dependent inhibition (Fig 4B). Furthermore, inhibition by the LuxO-R+linker construct was progressively more potent at lower ATP concentrations. These observations are consistent with the idea that the ATP substrate and the R-C linker compete for binding to the C domain active site, as implied by our crystal structures. As controls, we assayed the ability of the D60E phosphomimetic mutant of LuxO-R+linker and of a LuxO-R construct lacking the linker to inhibit LuxO-C in trans. LuxO-R+linker (D60E) displayed only a modest reduction in inhibitory activity relative to LuxO-R+linker (Fig 4C), perhaps because the phosphomimetic mutation fails to fully recapitulate the phosphorylated state (as also suggested by the data in Fig 4A). The absence of the linker in LuxO-R resulted in a larger impairment in inhibitory activity; however, in trans inhibition of the C domain was not abolished entirely (Fig 4C). This result suggests that the R domain itself has a role in inhibiting the C domain, in agreement with the in vivo results for *V*. *cholerae* LuxO (G145E) discussed above (Fig 3).

### Comparison of the LuxO Structure to Active R Domain Structures

Receiver domains, such as the one in LuxO, are ubiquitous regulatory modules in prokaryotic two-component signaling pathways [25–28]. Despite the remarkable diversity of the effector domains to which they are attached, receiver domains appear universally to adopt the same two stereotypical conformations, “active” and “inactive.” The relative stability of these two conformations is determined by whether or not the key receiver domain aspartate residue (Asp 54/60/61 in *V*. *angustum*, *V*. *vulnificus*, and *V*. *cholerae*, respectively) is phosphorylated or unphosphorylated; phosphorylation strongly favors the active state. The level of aspartate phosphorylation is, in turn, determined by the kinase activities of cognate two-component sensor kinase receptors.

**Fig 6:**
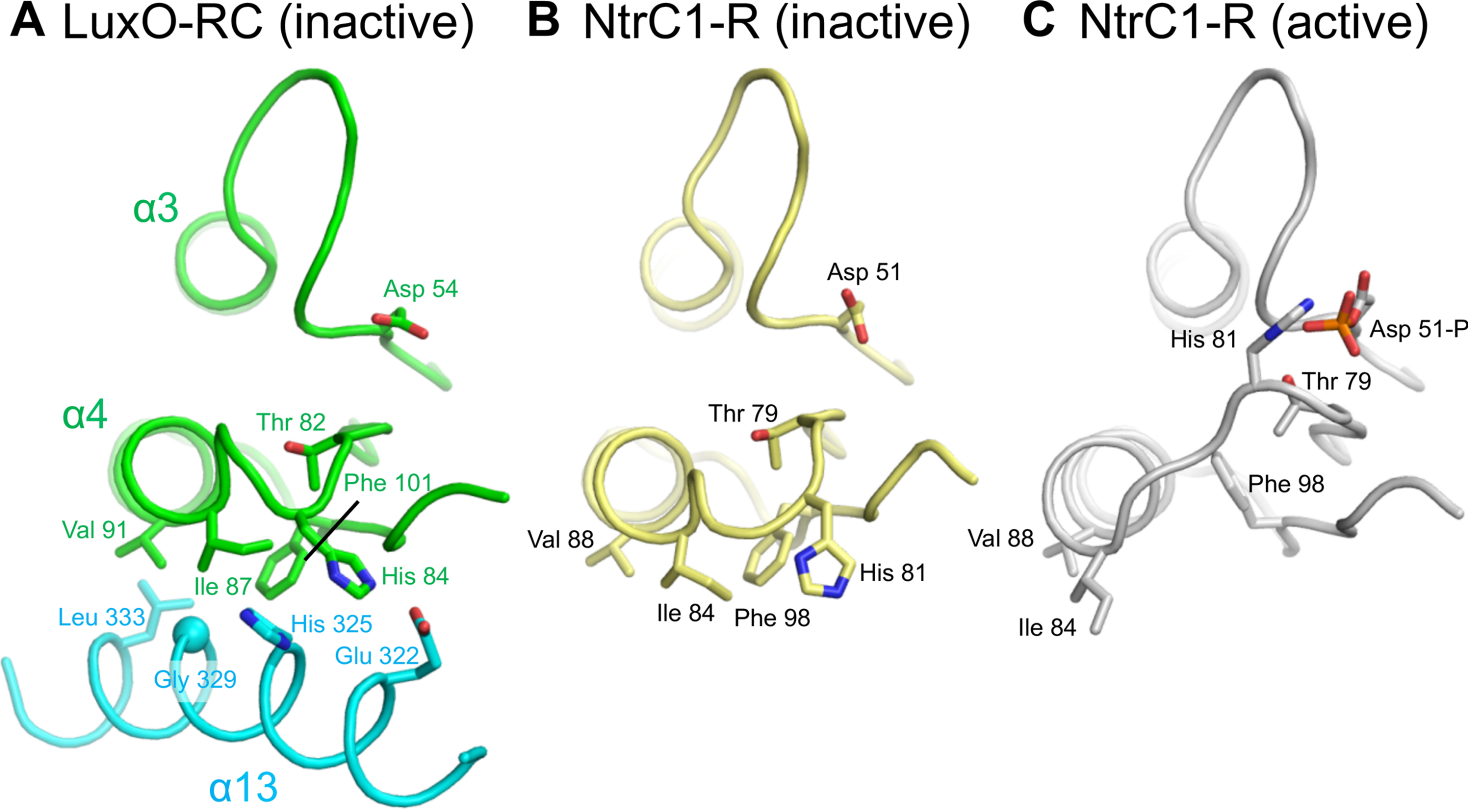
Receiver domain phosphorylation is predicted to disrupt interface II. (A) The *V*. *angustum* LuxO-RC structure shows how, in the unphosphorylated and autoinhibited conformation, helix α5 and several residues that precede it in the R domain (green) pack against α13 in the C domain (cyan). (B) The unphosphorylated R domain of the homologous protein NtrC1 (PDB code 1NY5) is similar to that of LuxO. (C) The phosphorylated R domain of NtrC1 (PDB code 1ZY2) undergoes the canonical conformational change from inactive to active. His 81 (His 84 in *V*. *angustum* LuxO) moves about 10 Å, which in LuxO would disrupt interactions with Glu 322 and His 325 (see panel A). NtrC Ile 84, Val 88, and Phe 101 (equivalent to *V*. *angustum* LuxO Ile 87, Val 91, and Phe 101, respectively) also move, which in LuxO-RC would disassemble a hydrophobic cluster stabilizing interface II.

The canonical conformational change associated with receiver domain phosphorylation [27,28] is predicted to have a large effect on residues that, in the LuxO-RC structure, lie in the interface between the R and C domains. The largest of these changes involves interface II (Fig 6A; see also Fig 2C). As an example, we consider the LuxO R domain residue His 84, which contributes to interface II through both salt bridge formation and π-stacking (with the LuxO C domain residues Glu 322 and His 325, respectively; Fig 6A). His 84 is located on the loop connecting β4 and α4 and, in homologous receiver domains, this loop is dramatically repositioned in response to receiver domain phosphorylation. For instance, in the R domain of the close LuxO homolog NtrC1 (for which both unphosphorylated and phosphorylated structures have been reported [22,23]), the corresponding His side chain moves ca. 10 Å upon phosphorylation (compare Fig 6B and 6C). This movement would break the salt bridge and π-stacking interactions that, in unphosphorylated LuxO, stabilize interface II. Additional conformational changes affecting α4 and β5 are expected to dismantle a hydrophobic cluster to which the LuxO R domain contributes Ile 87, Val 91, and Phe 101, further destabilizing interface II. Thus, the paradigmatic conformational change accompanying receiver domain phosphorylation is expected to disrupt the interaction between the LuxO R and C domains, providing a straightforward structural explanation for LuxO activation.

Once the phosphorylated R domain has “undocked” from the C domain, one might predict that the R-C linker would be more susceptible to limited proteolysis. Indeed, LuxO-RC (D60E) is more susceptible to proteolysis than is wild-type LuxO-RC (S2 Fig). Furthermore, wild-type LuxO-RC, the constitutively inactive mutant D60A, and the phosphomimetic mutant D60E all display similar sedimentation velocity ultracentrifugation profiles (Fig 5B–5D) consistent with hexamer formation. We do observe that activation (by D60E and/or BeF_3_) and/or nucleotide binding causes a broadening of the main peak (Fig 5B and 5D). This result might indicate a mixture of conformational states, or possibly of oligomerization states, that is not observed in the constitutively inactive D60A mutant. Further experiments will be required to determine the origin of this peak broadening.

### Inhibition of LuxO by AzaU

**Fig 7:**
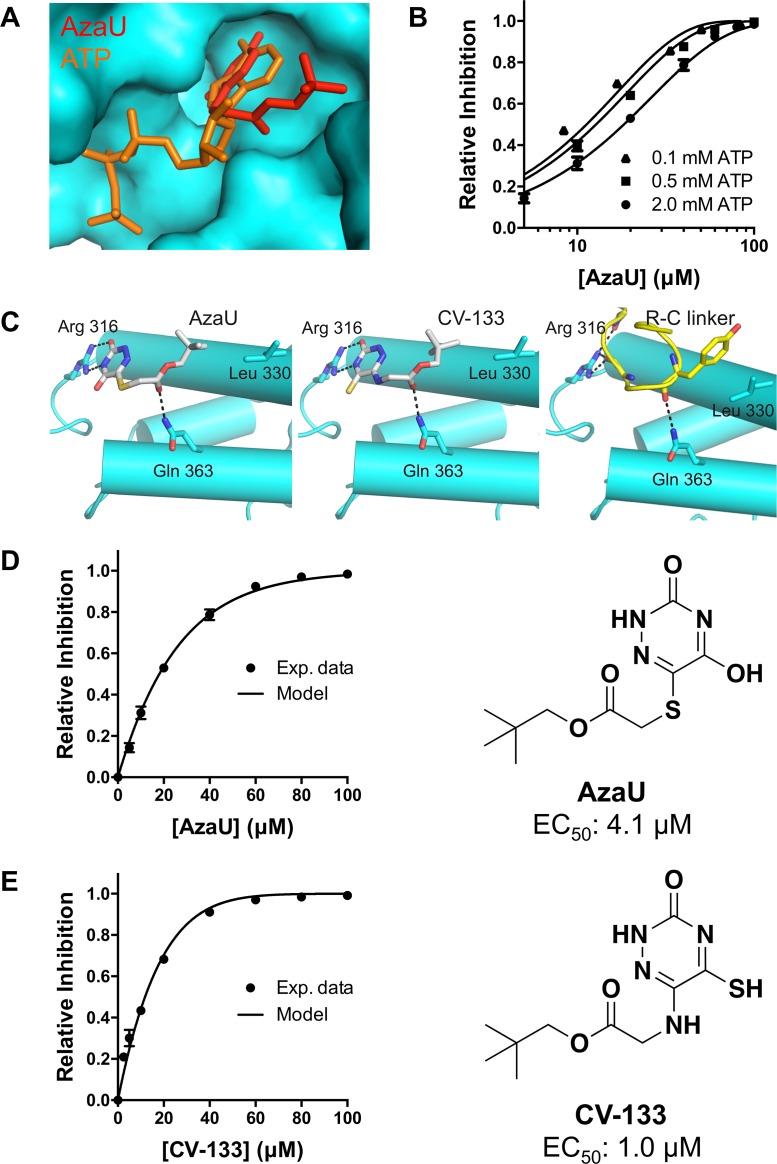
Structural and enzymatic characterization of LuxO inhibitors. (A) Comparing LuxO-C:ATP and LuxO-C:AzaU structures reveals that AzaU occupies a portion of the LuxO ATPase active site. (B) AzaU is a competitive inhibitor of *V*. *vulnificus* LuxO-C (100 μM), with higher substrate concentrations displaying reduced inhibition. Data are fit using the simple mathematical model described in the text. (C) AzaU, in its interaction with LuxO-C, mimics aspects of the R-C linker, including H-bonds with Arg 316 and Gln 363. (D and E) AzaU and CV-133 inhibition of *V*. *vulnificus* LuxO-C ([LuxO-C] = 100 μM, [ATP] = 2 mM) are shown, together with the inhibition curves predicted by the limiting-case model in which a single bound inhibitor molecule abolishes the activity of a LuxO oligomer. The best-fit values of the adjustable parameter, K, are given in the text. EC_50_ values were obtained using the *V*. *cholerae* assay (see Fig 3). Error bars represent standard deviation (*n* = 3); where no error bar is visible, it is smaller than the corresponding symbol. The data underlying panels B, D, and E can be found in S1 Data.

Previously, we reported the discovery and optimization of a set of 6-thio-5-azauracil compounds that activate vibrio quorum-sensing responses by inhibiting LuxO [8]. The most potent of these, with an EC_50_ in *V*. *cholerae* of 4.1 μM, was a tert-butyl analog previously called compound 12 and here renamed AzaU. To characterize the mechanism of AzaU inhibition of LuxO, we crystallized *V*. *angustum* LuxO-C in the absence of ligands (apo), in the presence of ATP, and in the presence of AzaU (S2 and S3 Tables). All three protein structures are highly similar (rms deviation < 0.5 Å). AzaU bound in the LuxO active site, with its modified uracil ring occupying the same region as the adenine ring of the ATP substrate, indicating that AzaU functions as a competitive inhibitor (Fig 7A). Indeed, consistent with this structural inference, ATPase assays using LuxO-C showed that AzaU inhibition is enhanced at lower ATP concentrations (Fig 7B), as expected for a competitive inhibitor. Strikingly, AzaU binds the LuxO active site in a manner that mimics the R-C linker, with both AzaU and the R-C linker forming H-bonds with Arg 316 and Gln 363 (Fig 7C).

In Fig 7D and 7E, the extent of LuxO-C inhibition is presented as a function of inhibitor concentration for AzaU and for a new, more potent inhibitor, CV-133 (EC_50_ = 1.0 μM). CV-133 closely resembles AzaU and, based on X-ray structural analysis (S3 Table), binds in an almost identical manner (Fig 7C). Sedimentation velocity analytical ultracentrifugation revealed that CV-133-bound LuxO-C is almost exclusively hexameric (Fig 5A), arguing strongly against the possibility that AzaU or CV-133 inhibits LuxO by causing ring dissociation. It is moreover evident from these plots that not every LuxO-C active site (present at 100 μM) needs to be occupied by an inhibitor molecule to obtain quantitative inhibition. Using mathematical modeling, we found that all of our LuxO-C inhibition data (Fig 7, panels B, D, and E) were well fit by a simple, limiting-case model in which a single inhibitor molecule is capable of inhibiting an entire LuxO-C ring (see S1 Modeling for details). Unfortunately, the relatively weak oligomerization of LuxO constructs has prevented us from measuring small molecule binding constants directly, for example, by isothermal titration calorimetry. In place of binding constants, our model therefore contains a single adjustable parameter K, representing the binding affinity of the inhibitor molecule relative to the substrate ATP (K = K_a_^AzaU^ / K_a_^ATP^ = K_d_^ATP^ / K_d_^AzaU^). The fitted K values for AzaU (K = 34 ± 2) and CV-133 (K = 268 ± 186) are roughly consistent with their relative EC_50_ values, with both comparisons implying that CV-133 binds substantially more tightly than AzaU to the LuxO active site. While we cannot rule out more complex models, all of our data can be well fit using a straightforward model—with a single adjustable parameter—in which only one inhibitor molecule is required to inhibit an entire LuxO hexamer.

## Discussion

Quorum sensing is a chemical communication process that bacteria use to control collective behaviors. Quorum sensing is crucial for virulence in pathogenic vibrios because it controls biofilm formation and virulence factor production [3,12,29]. Curiously, unlike in quorum-sensing bacteria that cause persistent infections, vibrios cause acute diseases, and quorum-sensing autoinducers repress biofilm formation and virulence factor production at high cell density [3]. Presumably, pro-quorum-sensing compounds that lock pathogenic vibrios into the high-cell-density quorum-sensing mode could be explored as therapies [7,8]. Multiple strategies readily come to mind, including agonizing the autoinducer receptors and modulating downstream components. Targeting the receptors could, however, prove difficult because multiple autoinducers are involved and they signal through distinct cognate receptors [2,3]. Thus, a combination strategy that simultaneously influences signaling via all of the receptors could be required. LuxO, by virtue of its key position in the quorum-sensing cascade, functioning downstream of the receptors to integrate sensory information emanating from all of the autoinducer-receptor pairs, is a particularly attractive candidate. Indeed, a Δ*luxO V*. *cholerae* strain is severely defective in the production of cholera toxin and the toxin coregulated pilus and is avirulent in a mouse model of infection [12]. Likewise, *V*. *cholerae* and other pathogenic vibrios treated with the LuxO inhibitor AzaU do not produce virulence factors [8]. Here, we explore the mechanisms underpinning the modulation of LuxO activity by phosphorylation and by small molecule inhibitors in an effort to forward our basic understanding of quorum-sensing signal transduction and to lay the groundwork for probing the promise of applications of quorum-sensing modulators.

Our studies demonstrate that the unphosphorylated R domain of LuxO inhibits the ATPase activity of the C domain by binding adjacent to the active site and stabilizing the R-C linker in a conformation that sterically occludes a substantial portion of the active site. To the best of our knowledge, this mode of AAA+ ATPase regulation has not previously been reported. Indeed, the closely related NtrC proteins are regulated in a completely different manner, as exemplified by *Aquifex aeolicus* NtrC1 [22] and *Salmonella typhimurium* NtrC [21]. NtrC1 in its inactive, unphosphorylated conformation is a homodimer, with both the R and C domains contributing to the intermonomer interface (S3 Fig). R domain phosphorylation drives a conformational change that disrupts the interface between the two R domains, permitting active oligomers to form [23]. NtrC exhibits a positive mode of regulation, in which the phosphorylated R domain is essential for the formation of active oligomers [21].

It has long been known that LuxO uses a negative mode of regulation, since deletion of its R domain is constitutively activating [18]. What is surprising is that LuxO has a unique mechanism of negative regulation not only compared to the AAA+ superfamily in general but compared to the closely related NtrC proteins in particular. In unphosphorylated NtrC1, the R domain helix α5 and the R-C linker form a single, long α-helix (S3 Fig). In unphosphorylated LuxO, by contrast, the R-C linker folds back onto α5, permitting the R domain to pack against the C domain of the same monomer and, in so doing, positioning a segment of the R-C linker so that it occupies the C domain active site. Despite the high homology between LuxO proteins and NtrC proteins, key amino acid differences appear to prevent NtrC proteins from adopting the inactive conformation we observe for LuxO. Indeed, in the two examples we tested (*V*. *cholerae* G145E and G333K; see Fig 3 and S1 Fig), exchanging the NtrC1 residue for the corresponding LuxO residue derepressed LuxO. These and a handful of other substitutions appear to be responsible for the drastic alteration in regulatory mechanism between LuxO and its most closely related homologs.

**Fig 8:**
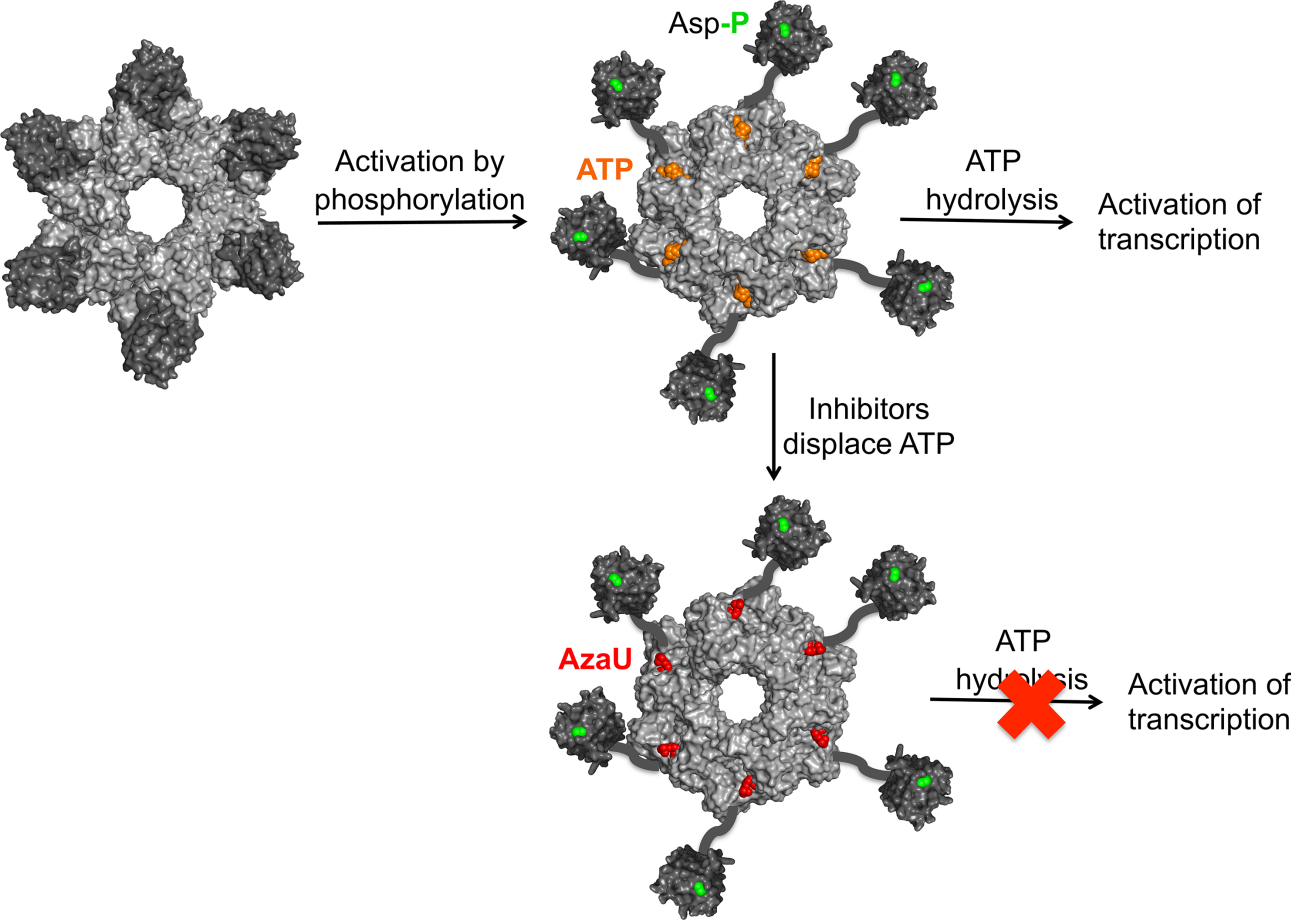
Proposed mechanism of LuxO activation and inhibition. Phosphorylation (green) activates LuxO by triggering a conformational change in the R domain that destabilizes the RC interface, dislocating the R-C linker from the LuxO ATP binding site and freeing the ATPase active site for catalysis (denoted by orange ATP). The location and dynamics of the phosphorylated R domains are unknown. Active LuxO uses the energy of ATP hydrolysis to open σ^54^-dependent promoters, converting them to transcriptionally active states. Active LuxO is also susceptible to competitive inhibition by AzaU or CV-133 (red). Binding of a single inhibitor molecule appears capable of inhibiting the entire ring.

The “front-to-front” dimers formed by unphosphorylated NtrC1 are incompatible with the formation of active rings [22]. By contrast, the autoinhibited LuxO monomers interact back-to-front in a ring-like helical arrangement that closely resembles, except for the occluded ATP binding site, the presumptive active state (Fig 8). This novel regulatory mechanism, by allowing preassembly of inactive oligomers at enhancer-like sequences upstream of σ^54^-dependent promoters, may confer a competitive advantage in signal transduction dynamics. Phosphorylation of pre-positioned LuxO oligomers could activate transcription of *qrr* genes and initiate the transition from the high-cell-density signaling state (LuxO inactive) to the low-cell-density signaling state (LuxO active), more rapidly than if LuxO employed an NtrC1 type of regulation. Since the low-cell-density state of LuxO is associated with superior colonization and infection of the host, an especially rapid adaptation might thereby confer a selective evolutionary advantage.

We found that the inhibitor molecules AzaU and CV-133, both derivatives of a small-molecule screening hit [8], function as competitive inhibitors of LuxO (Fig 8). AzaU had previously been reported to be an uncompetitive inhibitor based on Lineweaver-Burk analysis [8]. This analytic method assumes, however, that an inhibitor is capable of inhibiting only the monomer to which it is bound, whereas we now find that a single inhibitor molecule may be capable of inhibiting an entire oligomer. This new finding furthermore implies that there must be communication between LuxO subunits in the process of ATP hydrolysis and that the LuxO hydrolysis mechanism cannot, therefore, be entirely stochastic. Partially concerted mechanisms have been proposed for various AAA+ ATPases and for bEBPs, including NtrC1 [30] and PspF [31]. These intermediate mechanisms assume that monomers are heterogeneously occupied during ATP hydrolysis—some bound to ATP, others to ADP and/or unliganded—which is consistent with structural evidence for asymmetric states [15,17,32]. Nonetheless, it remains unclear how a single AzaU or CV-133 inhibitor molecule inhibits an entire LuxO oligomer. Our crystal structures do not reveal any substantial conformational changes associated with AzaU, CV-133, or ATP binding, possibly because of constraints imposed by the crystal lattice. In particular, the conformation of the GAFTGA loop, implicated in σ^54^ binding, is unaffected by either inhibitors or ATP, adopting in all cases a conformation similar to that of ADP-bound NtrC1 [17,22,30]. Furthermore, our analytical ultracentrifugation experiments show no evidence of a change in oligomerization state (Fig 5A). Further analysis will therefore be needed to distinguish among possible mechanisms.

Finally, AzaU inhibits *V*. *cholerae* LuxO without affecting cell growth, which—because vibrios possess multiple AAA+ ATPases that underpin central biological processes—suggests that AzaU displays high specificity for LuxO. Nonetheless, higher specificity may ultimately be achieved by identifying and optimizing allosteric inhibitors. Alternatively, extending the idea that AzaU recapitulates some of the key interactions that stabilize the R-C linker in the ATPase active site, it may prove possible to engineer potent and selective second-generation inhibitors that capture even more of these interactions.

## Materials and Methods

### Protein Production and Purification

DNA encoding various LuxO constructs was amplified by PCR from genomic DNA and cloned into pET21b or pET28b for the production of His_6_-tagged fusion proteins (S1 Table). The His_6_ tag was placed at the C-terminus for all constructs except the construct containing the R domain and R-C linker of *V*. *vulnificus* LuxO used in Fig 4B and 4C; in that case, the tag was located at the N-terminus. For protein purification, *Escherichia coli* BL21(DE3) cells containing one of the expression plasmids described above were grown at 37°C to OD_600_ ~ 0.6, followed by induction with 0.1 mM IPTG at 16°C for 16–18 h. Cells, collected by centrifugation at 5,000 g for 15 min, were resuspended in lysis buffer (50 mM Tris-HCl, pH 8.0, 300 mM NaCl, 20% [v/v] glycerol) and lysed using an Emulsiflex-C5 homogenizer (Avestin). The resulting lysates were supplemented with 10 μg/ml DNase (Roche), 4 mM β-mercaptoethanol, and protease inhibitor cocktail tablets (Roche), incubated for 30 min at 4°C, and clarified by centrifugation at 30,000 g for 1 h. The His_6_-LuxO constructs were purified from the clarified lysates by binding to His60 Ni Superflow resin (ClonTech), which was then washed using lysis buffer containing 20 mM imidazole and 4 mM β-mercaptoethanol. Proteins were eluted using lysis buffer containing 500 mM imidazole and 4 mM β-mercaptoethanol and further purified using a Superdex 200 HR 10/30 column (GE Healthcare) pre-equilibrated in 20 mM Tris-HCl, pH 8.0, 150 mM NaCl, 1 mM dithiothreitol (DTT), 10% (v/v) glycerol. All proteins except LuxO-R eluted as broad peaks, showing that different oligomeric states of LuxO were present. After concentration, protein stocks (approximately 15 mg/ml protein) were stored at −80°C.

### Protein Crystallization

*V*. *angustum* LuxO-C (residues 141–387) crystals were grown at 20°C using the hanging drop vapor diffusion method with a 1∶1 (v/v) mixture of protein (5 mg/ml) and precipitant solution (3.2 M ammonium acetate, 0.1 M HEPES, pH 7.5). LuxO-C:AzaU, LuxO-C:CV-133, and LuxO-C:ATP crystals were grown under the same conditions except for the precipitant solutions used (3.0 M ammonium acetate, 0.1 M Tris-HCl, pH 8.0 for the AzaU complex; 1.4 M ammonium sulfate, 0.1 M HEPES, pH 7.5 for the CV-133 complex; and 0.25 M NaCl, 25% [w/v] PEG3350, 0.1 M HEPES, pH 7.5 for the ATP complex) and the addition of ligand. AzaU and CV-133 were added in 1.2-fold molar excess relative to LuxO-C, whereas ATP was added at 10 mM (without Mg^2+^ to prevent hydrolysis). Unit cell dimensions for LuxO-C crystals were a = b = 76 Å, c = 82 Å, α = β = 90.0°, γ = 120.0° in space group P6_1_, with one monomer in the asymmetric unit. *V*. *angustum* LuxO-RC (residues 1–387, plus a C-terminal His_6_ tag) was crystallized as above, except that the protein concentration was 6 mg/ml and the precipitant solution contained 2 M ammonium sulfate, 0.1 M sodium acetate, pH 5.5. The size of the LuxO-RC crystals was increased by streak seeding. Unit cell dimensions were a = b = 116 Å, c = 69 Å, α = β = 90.0°, γ = 120.0° in space group P6_1_, with one monomer in the asymmetric unit.

### Structure Determination and Refinement

X-ray data for LuxO:AzaU were collected using beamline X29 of the National Synchrotron Light Source (NSLS) at Brookhaven National Laboratory. X-ray data for LuxO-C (native), LuxO-C:ATP, and LuxO-RC were collected using beamline F-1 at the Cornell High-Energy Synchrotron Source (CHESS). Data for LuxO-C:CV-133 were collected using a home source (Rigaku RU-H3R X-ray generator, Rigaku RAXIS-IV++ detector, Xenocs FOX 2D multilayer optics). In each case, crystals were flash-cooled to 100 K and maintained at that temperature for the duration of data collection; as cryoprotectants, we used 20% (v/v) ethylene glycol (LuxO-C) or 15% (v/v) ethylene glycol (LuxO-RC). The structure of LuxO-C:AzaU was determined by the method of molecular replacement using the program PHASER [33] with a model of the catalytic domain from *A*. *aeolicus* NtrC1 (PDB entry 1NY5 [22]). The structures of LuxO-C, LuxO-C:ATP, and LuxO-C:CV-133 were obtained by rigid-body refinement from the LuxO-C:AzaU structure. The structure of LuxO-RC was determined by the molecular replacement method using the LuxO-C:AzaU structure as the model for the catalytic domain model and the receiver domain from *A*. *aeolicus* NtrC1 (PDB entry 1NY5) as the model for the receiver domain. Structures were manually rebuilt using the program COOT [34] and refined using Phenix.refine [35]. Data collection and structure refinement statistics are provided in S2 and S3 Tables.

The current model of apo-LuxO-C contains residues 141–392, including residues from the C-terminal His_6_ tag, plus acetate ions from the crystallization solution. The current model of LuxO-C:AzaU contains residues 141–392, including residues from the C-terminal His_6_ tag, plus acetate ions from the crystallization solution. The current model of LuxO-C:CV-133 contains residues 141–393, including residues from the C-terminal His_6_ tag, plus sulfate and HEPES ions from the crystallization solution and an ethylene glycol molecule from the cryoprotectant condition. CV-133 is modeled with a partial occupancy of 0.68. The current model of LuxO-C:ATP contains residues 141–393, including residues from the C-terminal His_6_ tag, a HEPES ion from the crystallization solution, and an ethylene glycol molecule from the cryoprotectant condition. ATP is modeled with a partial occupancy of 0.84. The current model of LuxO-RC contains residues 3–388, including residues from the C-terminal His_6_ tag, sulfate ions from the crystallization solution, and an ethylene glycol molecule from the cryoprotectant condition. Loops corresponding to residues 124–126 in the R-C linker and residues 221–223 were not placed in the model because of insufficient ordered electron density.

### LuxO Mutant Construction and Bioluminescence Assays

Site-directed mutagenesis of the wild-type *V*. *cholerae luxO* gene carried on plasmid pEVS143 was used to introduce the following mutations: F108A, F108W, V120E, G145E, G333A, and G333K. Primers used for the mutagenesis are provided in S4 Table. The LuxO D61A mutation was also introduced into wild-type LuxO and into each of the single mutants listed above. The resulting single and double LuxO-containing mutant plasmids were conjugated into the *V*. *cholerae* bioluminescent reporter strain AH261 (*V*. *cholerae* Δ*luxO* Δ*lacZ*:*hapR*/pBB1). Plasmid pEVS143 carrying LuxO D61E was introduced into *V*. *cholerae* AH261 and used as a positive control.

Overnight cultures from single colonies of the *V*. *cholerae* reporter strain carrying the various LuxO alleles were grown in LB medium supplemented with tetracycline (10 mg/ml) and kanamycin (50 mg/ml) at 37°C. Cultures were back-diluted to an OD_600_ = 0.001 with sterile medium. Following a 6-h incubation at 37°C with shaking, OD_600_ was measured using a DU800 spectrophotometer. Bioluminescence was measured in triplicate on an Envision Multilabel Reader and normalized to that of the wild-type reporter strain.

### ATPase Assays

ATPase assays were performed using a standard NADH-coupled ATPase assay as described previously [8] with minor modifications: 100 mM K-HEPES, pH 7.4 was used in place of sodium phosphate buffer, and BSA (250 μg/ml) and β-mercaptoethanol (4 mM) were added. All assays were performed at 23°C using a Beckman Coulter DU800 spectrophotometer with a path length of 1.0 cm. In each trial, the absorbance of NADH at 340 nm was measured at 1.5 s intervals for 15 s (or until the NADH was depleted). DMSO was added to a final concentration of 4% in all inhibitor trials.

### Analytical Ultracentrifugation

Sedimentation velocity experiments were performed using a Beckman model ProteomeLab XL-A instrument equipped with an An-Ti60 rotor. Sedimentation of the boundary was measured at 42,000 rpm at 20°C using a step size of 0.003 cm and a delay time of 0 s and collecting between 70–100 scans. Samples were monitored at 250 nm, 280 nm, and 290 nm, with a requirement that the initial absorbance be below 1.5. For samples containing the competitive inhibitor CV-133, samples were also monitored at 335 nm. *V*. *vulnificus* LuxO-C and LuxO-RC constructs were dialyzed overnight at 4°C toward 20 mM Tris-HCl, pH 8.0, 150 mM NaCl, 5 mM MgCl_2_, and 1 mM DTT and stock solutions were diluted in dialysis buffer plus 4% DMSO to reach 60–100 μM monomer concentration. Experiments with CV-133 contained 60 μM LuxO-C and 120 μM CV-133. Experiments with ADP:BeF_3_ contained 100 μM LuxO-C or LuxO-RC, 150 μM ADP, 750 μM BeSO_4_, and 3.76 mM NaF. Temperature-corrected partial specific volumes, densities, and viscosities were calculated using Sednterp (v. 1.09) [36]. Model-independent continuous, c(s), distribution analysis for determining sample heterogeneity was performed using Sedfit (v. 14.4d) [37]. Regularization of the distribution by the maximum entropy method was applied with the parameter α constrained to a value of 0.95. Molar masses and diffusion coefficients were determined using DCDT+ (v. 2.4.0) [38].

Pilot experiments using LuxO-C and LuxO-RC (D60E) over a wide range of concentrations (15–230 μM) in 20 mM Tris-HCl, pH 8.0, 150 mM NaCl, 1 mM DTT indicated a self-associating system with three components: monomers; intermediates, which are likely dimers; and hexamers. LuxO-C is driven by the presence of CV-133 (a competitive inhibitor) or ADP:BeF_3_ (a substrate/transition state analog) into a predominantly hexameric state with the major peak at approximately 7.4 S (Fig 5A). The corresponding s(_20,w_) value coupled with a diffusion coefficient D(_20,w_) of 3.8 x 10^−7^ cm^2^ s^-1^ gives a mass of 174 ± 2 kDa, in excellent agreement with the theoretical mass of a LuxO-C hexamer (173 kDa). Where monomers and/or other subhexameric species are present, the apparent sedimentation rate can be suppressed by the Johnston-Ogston effect [39], which may in turn account for the lower apparent mobility of LuxO-C in the absence of inhibitor or substrate (Fig 5A). In further pilot experiments, LuxO-RC (D60E) c(s) distributions also displayed a concentration dependence, with a monomer peak at about 4.5 S, a dimer peak at about 6.7 S, and a hexamer peak at about 9.2 S. In the presence of ADP:BeF_3_, LuxO-RC constructs displayed changes in these distributions consistent with shifts in the equilibrium toward the hexameric state. In all of the experiments shown in Fig 5B–5D, the molar mass (as calculated by DCDT+) of the predominant state fell within the range 262–275 kDa. This value is in excellent agreement with the predicted molecular weight of a LuxO-RC hexamer (269 kDa). We conclude that both inactive and active LuxO-C and LuxO-RC form hexamers in a concentration-dependent manner. The position of this equilibrium can be substantially affected by the presence of ADP:BeF3 and/or CV-133, as observed for LuxO-RC (WT), LuxO-RC (D60E), and LuxO-C (Fig 5).

### Chemical Synthesis and Analytical Methods

The synthesis and analysis of AzaU was reported previously [8]. The synthesis and analysis of CV-133 is provided in S1 Methods.

## Supporting Information

S1 Data Excel file containing in separate sheets the numerical data for Figs 3, 4A–4C, 7B, 7D and 7E. (XLSX)

S1 Fig Multiple sequence alignment of vibrio LuxO proteins and selected NtrC proteins. R (green) and C (cyan) domains, and the R-C linker (yellow), are indicated. Each protein also contains a C-terminal DNA-binding domain (not depicted). Cylinders and arrows represent α-helices and β-strands, respectively, in our X-ray structure of LuxO-RC. Residues altered by site-directed mutagenesis of *V*. *cholerae* LuxO (see Fig 3) are indicated by boxes.
(PDF)

S2 Fig Limited proteolysis of *V*. *vulnificus* LuxO-RC (wild type) and LuxO-RC (D60E). LuxO-RC proteins were digested with (A) subtilisin, (B) chymotrypsin, or (C and D) proteinase K at 23°C. Digestion times and protease dilutions (based on 5 mg/ml stock solutions) are indicated.
(PDF)

S3 Fig Structural comparison of autoinhibited NtrC1 and LuxO. Shown are (A) the inactive dimer of NtrC1 (PDB entry 1NY5), (B) an isolated monomer from the same structure, and (C) the monomer structure of LuxO-RC. The LuxO residues mutated in Fig 3, and the corresponding residues in NtrC1, are indicated as spheres using the color code employed in Fig 3.
(PDF)

S1 Methods Supplementary Materials and Methods. (PDF)

S1 Modeling Mathematical modeling of competitive inhibition. (PDF)

S1 Table Vibrio constructs tested. pET21b-based expression plasmids were generated from genomic DNA using the primers provided here.
(PDF)

S2 Table Crystallographic data, phasing and refinement statistics: Apo structures. (PDF)

S3 Table Crystallographic data, phasing and refinement statistics: Liganded structures. (PDF)

S4 Table *V*. *cholerae* LuxO site-directed mutagenesis primer sequences. Lowercase letters indicate the altered nucleotide(s).
(PDF)

## Abbreviations

bEBP: bacterial enhancer-binding protein
C: central
CHESS: Cornell High-Energy Synchrotron Source
D: DNA-binding
NSLS: National Synchrotron Light Source
R: receiver
rms: root mean square
sRNA: small RNA
