# Supplementary material for: Structure, Regulation, and Inhibition of the Quorum-Sensing Signal Integrator LuxO
Source: PLoS Biol. 2016 May 24;14(5):e1002464. doi: 10.1371/journal.pbio.1002464 (PMC4878744; doi:10.1371/journal.pbio.1002464)
Supplement: S1 Fig — R (green) and C (cyan) domains, and the R-C linker (yellow), are indicated. Each protein also contains a C-terminal DNA-binding domain (not depicted). Cylinders and arrows represent α-helices and β-strands, respectively, in our X-ray structure of LuxO-RC. Residues altered by site-directed mutagenesis of V. cholerae LuxO (see Fig 3) are indicated by boxes. (PDF) [file pbio.1002464.s002.pdf]

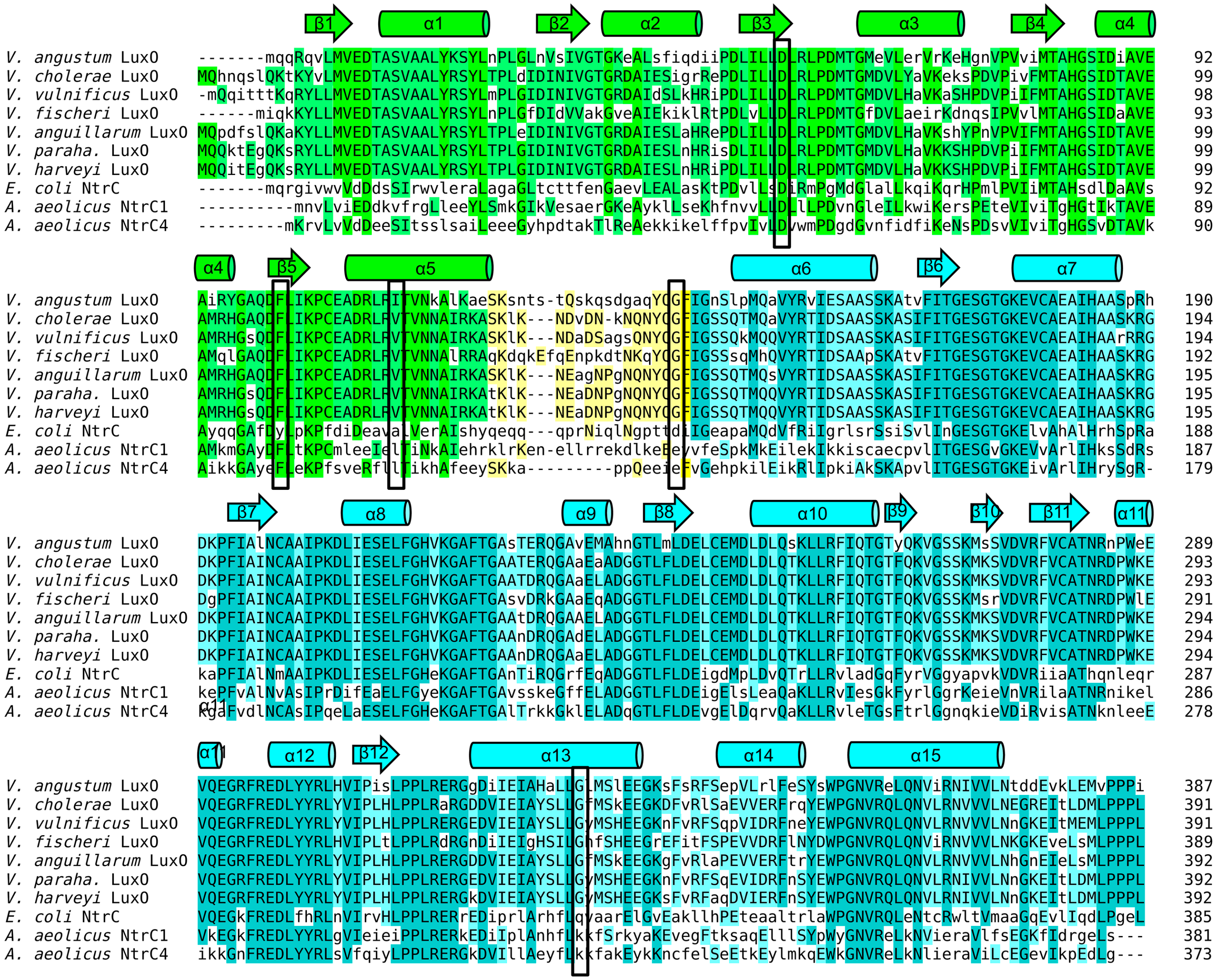

β1 α1 β2 α2 β3 α3 β4 α4
V. angustum LuxO 92
V. cholerae LuxO 99
V. vulnificus LuxO 98
V. fischeri LuxO 93
V. anguillarum LuxO 99
V. paraha. LuxO 99
V. harveyi LuxO 99
E. coli NtrC 92
A. aeolicus NtrC1 89
A. aeolicus NtrC4 90
α4 β5 α5 α6 β6 α7
V. angustum LuxO 190
V. cholerae LuxO 194
V. vulnificus LuxO 194
V. fischeri LuxO 192
V. anguillarum LuxO 195
V. paraha. LuxO 195
V. harveyi LuxO 195
E. coli NtrC 188
A. aeolicus NtrC1 187
A. aeolicus NtrC4 179
β7 α8 α9 β8 α10 β9 β10 β11 α11
V. angustum LuxO 289
V. cholerae LuxO 293
V. vulnificus LuxO 293
V. fischeri LuxO 291
V. anguillarum LuxO 294
V. paraha. LuxO 294
V. harveyi LuxO 294
E. coli NtrC 287
A. aeolicus NtrC1 286
A. aeolicus NtrC4 278
α11 α12 β12 α13 α14 α15
V. angustum LuxO 387
V. cholerae LuxO 391
V. vulnificus LuxO 391
V. fischeri LuxO 389
V. anguillarum LuxO 392
V. paraha. LuxO 392
V. harveyi LuxO 392
E. coli NtrC 385
A. aeolicus NtrC1 381
A. aeolicus NtrC4 373
