# Supplementary figures and images for: Structure, Regulation, and Inhibition of the Quorum-Sensing Signal Integrator LuxO
Source: PLoS Biol. 2016 May 24;14(5):e1002464. doi: 10.1371/journal.pbio.1002464 (PMC4878744; doi:10.1371/journal.pbio.1002464)

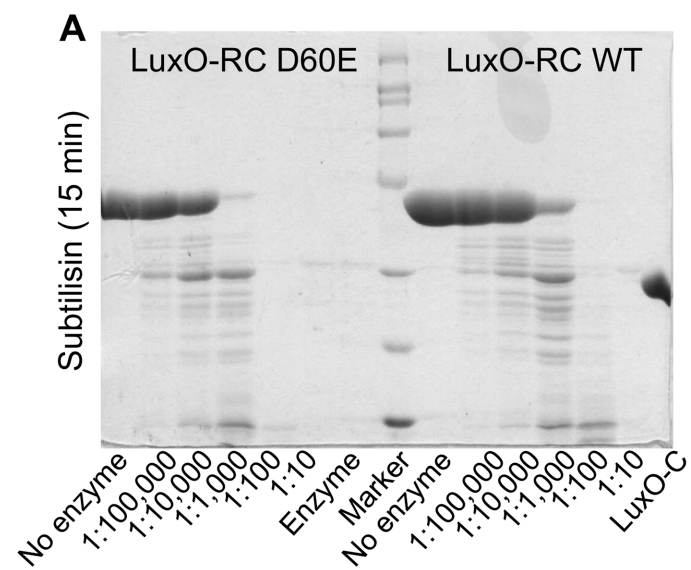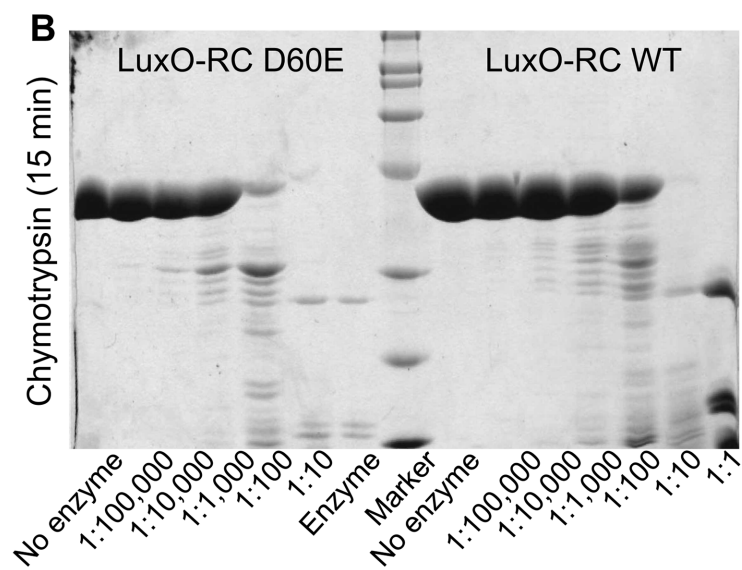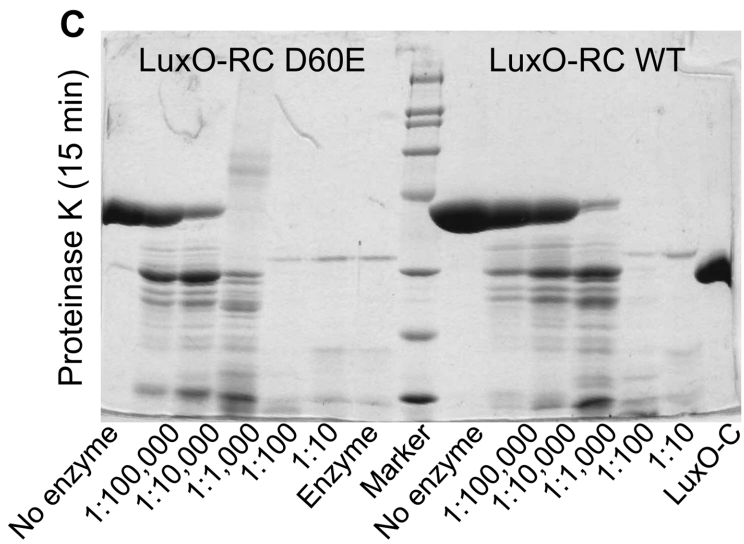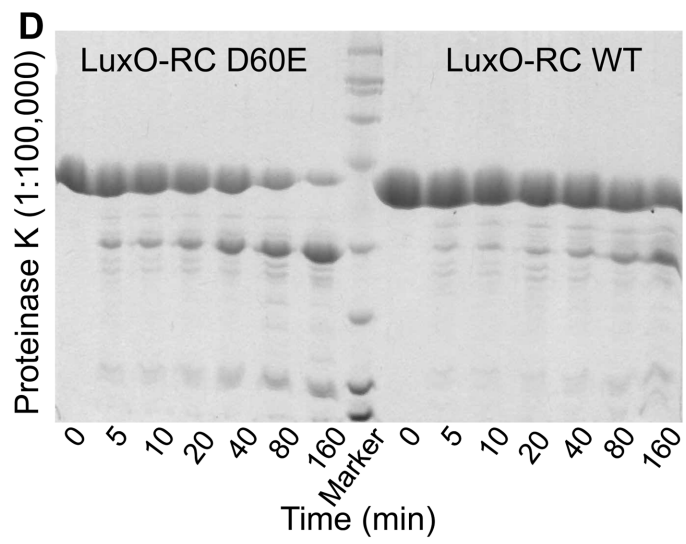

Supplement: S2 Fig — LuxO-RC proteins were digested with (A) subtilisin, (B) chymotrypsin, or (C and D) proteinase K at 23°C. Digestion times and protease dilutions (based on 5 mg/ml stock solutions) are indicated. (PDF) [file pbio.1002464.s003.pdf]

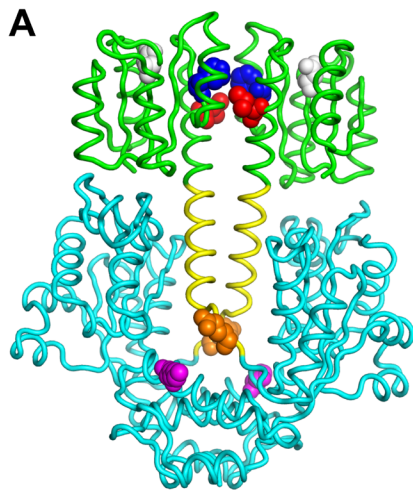

NtrC1 dimer

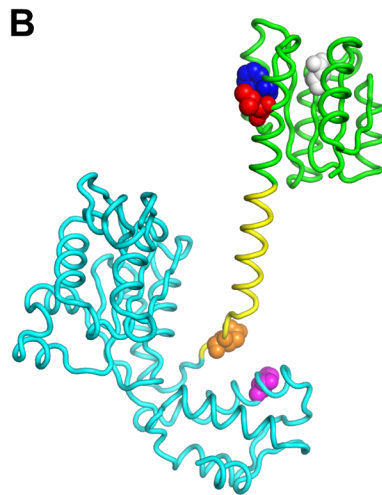

NtrC1 monomer

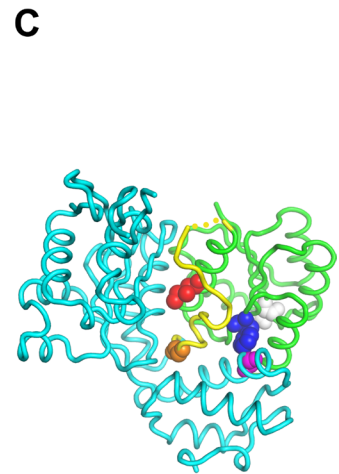

LuxO monomer

Supplement: S3 Fig — Shown are (A) the inactive dimer of NtrC1 (PDB entry 1NY5), (B) an isolated monomer from the same structure, and (C) the monomer structure of LuxO-RC. The LuxO residues mutated in Fig 3, and the corresponding residues in NtrC1, are indicated as spheres using the color code employed in Fig 3. (PDF) [file pbio.1002464.s004.pdf]
