# Supplementary material for: Structure, Regulation, and Inhibition of the Quorum-Sensing Signal Integrator LuxO
Source: PLoS Biol. 2016 May 24;14(5):e1002464. doi: 10.1371/journal.pbio.1002464 (PMC4878744; doi:10.1371/journal.pbio.1002464)
Supplement: S1 Methods — (PDF) [file pbio.1002464.s005.pdf]

Supplementary Materials and Methods

Limited proteolysis

Purified *V. vulnificus* LuxO-RC (wild-type and D60E) were diluted to 1.6 mg/ml (36 μM) in 20 mM Tris pH 8.0, 150 mM NaCl, 1 mM DTT, 10% [v/v] glycerol. Proteolytic digestion was initiated by mixing equal volumes of protein and protease (diluted from a 5 mg/ml stock solution as indicated), followed by incubation at 23°C. Reactions were terminated by adding SDS-PAGE loading buffer and immediately boiling the samples for 3 min. The digestion products were separated using SDS-PAGE.

Synthesis and analysis of CV-133

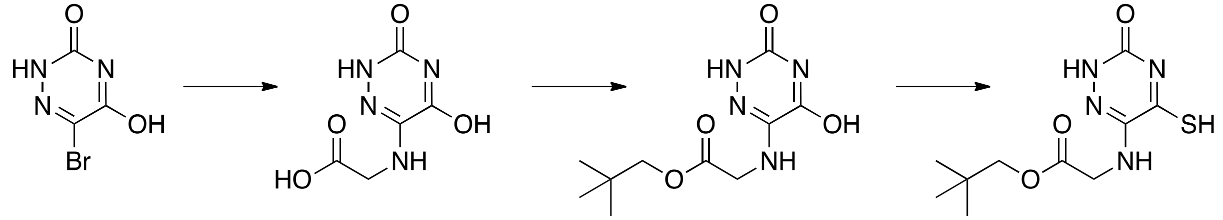

bromoazauracil **CV-96** **CV-102** **CV-133**

CV-96. To bromoazauracil (1.00 g, 5.2 mmol) was added glycine (0.780 g, 10.0 mmol), CuI (0.099 g, 0.52 mmol), and K_3PO_4 (3.86 g, 18.2 mmol). The mixture was diluted with water (5.2 mL) and deanol (1.56 mL). The mixture was heated to 90 °C for 3 d. Then, the mixture was cooled to rt. Concentrated HCl was added dropwise until pH ~5, and the mixture was stirred at rt for 1 h. The resultant precipitate was collected *via* vacuum filtration and washed with cold EtOH. The precipitate was dried further *in vacuo* to afford a colorless solid (0.740 g, 76%): ^{1}H NMR (500 MHz, D_2O) δ 3.82 (s, 2H); ^{13}C NMR (126 MHz, D_2O) δ 175.9, 155.0, 150.3, 143.7, 43.6; HRMS (ESI) *m/z* calcd for $C_5H_7N_4O_4$ $(M+H)^+$ 187.04618, found 187.04625.

CV-102. To neopentanol (5.64 g, 64.0 mmol) in CH_2Cl_2 (3.2 mL) cooled to 0 °C was added $SOCl_2$ (0.46 mL, 6.4 mmol) and then **CV-96** (0.600 g, 3.2 mmol). The mixture was warmed to rt and then to 100 °C for 24h. The solution was cooled to rt and excess solvent was removed *in vacuo*. The resultant residue was diluted with EtOAc (25 mL) and stirred for 15 min. The resultant precipitate was collected via filtration and dried *in vacuo* to afford a colorless solid (0.560 g, 68%). Additional product was isolated from the filtrate; the filtrate was concentrated *in vacuo* and purified by column chromatography (10:90, MeOH:CH_2Cl_2) to afford the product as a colorless solid (0.229 g, 28% (96% total yield)): ^{1}H NMR (500 MHz, DMSO-d_6) δ 11.90 (s, 1H), 11.10 (s, 1H), 6.96 (t, J = 6.5 Hz, 1H), 3.86 (d, J = 6.5 Hz, 2H), 3.74 (s, 2H), 0.85 (s, 9H); ^{13}C NMR (126 MHz, DMSO-d_6) δ 169.9, 154.3, 148.7, 142.3, 73.2, 42.5, 31.2, 26.1; HRMS (ESI) *m/z* calcd for $C_{10}H_{17}N_4O_4$ $(M+H)^+$ 257.12443, found 257.12379.

CV-133. To **CV-102** (0.050 g, 0.20 mmol) in pyridine (3.9 mL) was added phosphorus pentasulfide (0.047 g, 0.21 mmol). The mixture was heated to reflux for 6 h. After cooling to rt, the solution was decanted and concentrated *in vacuo*. To the resultant residue was added water (10 mL), and the mixture was boiled for 10 min. After cooling to rt, the resultant precipitate was collected by filtration. The solid was dissolved in EtOAc (10 mL), dried over Na_2SO_4, filtered, and concentrated *in vacuo*. The product was purified by column chromatography (40:60,

EtOAc:hexanes) to afford a pale yellow solid (0.037 g, 65%): ^{1}H NMR (500 MHz, DMSO-d_6) δ 13.53 (s, 1H), 11.71 (s, 1H), 6.79 (t, *J* = 6.4 Hz, 1H), 3.94 (d, *J* = 6.4 Hz, 2H), 3.74 (s, 2H), 0.85 (s, 9H); ^{13}C NMR (126 MHz, DMSO-d_6) δ 179.1, 169.9, 145.5, 144.6, 73.2, 43.4, 31.2, 26.1; HRMS (ESI) *m/z* calcd for $C_{10}H_{17}N_4O_3S$ $(M+H)^+$ 273.10159, found 273.10187.
