# Supplementary material for: Structure, Regulation, and Inhibition of the Quorum-Sensing Signal Integrator LuxO
Source: PLoS Biol. 2016 May 24;14(5):e1002464. doi: 10.1371/journal.pbio.1002464 (PMC4878744; doi:10.1371/journal.pbio.1002464)
Supplement: S1 Modeling — (PDF) [file pbio.1002464.s006.pdf]

Mathematical modeling of competitive inhibition

k_{cat} values were converted to relative inhibition according to the following formula, in which k_{cat}^{max} represents enzyme activity in the absence of inhibitor:

$$\text{relative inhibition} = 1 - \frac{k_{cat}^{observed}}{k_{cat}^{max}}$$

As a starting point, we assume independent and equivalent inhibitor binding sites. The probability of a given site being occupied, α, equals the ratio of the number of occupied inhibitor binding sites to the total inhibitor binding sites, or equivalently the ratio of their concentrations [PI] and P_{tot}. To derive an expression for α = [PI] / P_{tot} , we assume (based on our inhibitor-bound crystal structures and on the LuxO-C inhibition data in Fig. 7B) that AzaU and CV-133 are competitive inhibitors. Then, the relevant equilibrium dissociation constants are defined as:

$$K_d^{inhibitor} = \frac{[P][I]}{[PI]} = \frac{(P_{tot} - [PI] - [PS])(I_{tot} - [PI])}{[PI]} \qquad (\mathbf{a})$$

$$K_d^{ATP} = \frac{[P][S]}{[PS]} = \frac{(P_{tot} - [PI] - [PS])(S_{tot} - [PS])}{[PS]}$$

where S_{tot} and I_{tot} are the total concentrations of the substrate (ATP) and the inhibitor, respectively, and [PS] and [PI] are the concentrations of substrate- and inhibitor-bound LuxO-C monomers.

Taking the ratio yields:

$$\frac{K_d^{inhibitor}}{K_d^{ATP}} = \frac{(I_{tot} - [PI])[PS]}{(S_{tot} - [PS])[PI]}$$

which, upon rearrangement, gives:

$$[PS] = \frac{\left(K_d^{inhibitor}\right)(S_{tot})[PI]}{\left(K_d^{ATP}\right)(I_{tot}) + \left(K_d^{inhibitor} - K_d^{ATP}\right)[PI]} \qquad (\mathbf{b})$$

For a potent inhibitor, $K_d^{inhibitor} \ll K_d^{ATP}$, allowing **(b)** to be simplified:

$$[PS] \approx \frac{\left(K_d^{inhibitor}\right)(S_{tot})[PI]}{\left(K_d^{ATP}\right)(I_{tot}) + \left(-K_d^{ATP}\right)[PI]} = \frac{\left(K_d^{inhibitor}\right)(S_{tot})[PI]}{\left(K_d^{ATP}\right)(I_{tot} - [PI])} \qquad (\mathbf{c})$$

Returning to equation **(a)**, and rearranging, yields:

$$[PI]^2 - \left(P_{tot} + I_{tot} + K_d^{inhibitor}\right)[PI] + (P_{tot})(I_{tot}) = (I_{tot} - [PI])[PS] \quad (\mathbf{d})$$

into which [PS], as defined in equation **(c)**, can be substituted:

$$[PI]^2 - \left(P_{tot} + I_{tot} + K_d^{inhibitor}\right)[PI] + (P_{tot})(I_{tot}) = (I_{tot} - [PI])(\frac{\left(K_d^{inhibitor}\right)(S_{tot})[PI]}{\left(K_d^{ATP}\right)(I_{tot} - [PI])}$$

$$[\mathrm{PI}]^2-\left(\mathrm{P_{tot}}+\mathrm{I_{tot}}+\mathrm{K_d^{inhibitor}}\right)[\mathrm{PI}]+(\mathrm{P_{tot}})(\mathrm{I_{tot}})=\left(\frac{\left(\mathrm{K_d^{inhibitor}}\right)(\mathrm{S_{tot}})}{\left(\mathrm{K_d^{ATP}}\right)}\right)[\mathrm{PI}]$$

$$[\mathrm{PI}]^2-\left(\mathrm{P_{tot}}+\mathrm{I_{tot}}+\mathrm{K_d^{inhibitor}}+\frac{\left(\mathrm{K_d^{inhibitor}}\right)}{\left(\mathrm{K_d^{ATP}}\right)}(\mathrm{S_{tot}})\right)[\mathrm{PI}]+(\mathrm{P_{tot}})(\mathrm{I_{tot}})=0$$

Since $\mathrm{P_{tot}}+\mathrm{I_{tot}}\gg\ \mathrm{K_d^{inhibitor}}$ (see below),

$$[\mathrm{PI}]^2-\left(\mathrm{P_{tot}}+\mathrm{I_{tot}}+\frac{\left(\mathrm{K_d^{inhibitor}}\right)}{\left(\mathrm{K_d^{\mathit{ATP}}}\right)}(\mathrm{S_{tot}})\right)[\mathrm{PI}]+(\mathrm{P_{tot}})(\mathrm{I_{tot}})=0$$

Solving for [PI] using the quadratic formula, and defining $\mathrm{K}=\mathrm{K_d^{ATP}}/\mathrm{K_d^{inhibitor}}$, yields:

$$[\mathrm{PI}]=\frac{\left(\mathrm{P_{tot}}+\mathrm{I_{tot}}+\frac{(\mathrm{S_{tot}})}{\mathrm{K}}\right)-\sqrt{\left(\mathrm{P_{tot}}+\mathrm{I_{tot}}+\frac{(\mathrm{S_{tot}})}{\mathrm{K}}\right)^2-4(\mathrm{P_{tot}})(\mathrm{I_{tot}})}}{2}$$

Thus, since α = [PI] / P_{tot} , we conclude that:

$$\alpha=\frac{\left(\mathrm{P_{tot}}+\mathrm{I_{tot}}+\frac{(\mathrm{S_{tot}})}{\mathrm{K}}\right)-\sqrt{\left(\mathrm{P_{tot}}+\mathrm{I_{tot}}+\frac{(\mathrm{S_{tot}})}{\mathrm{K}}\right)^2-4(\mathrm{P_{tot}})(\mathrm{I_{tot}})}}{2\mathrm{P_{tot}}}$$

We note that, in the above derivation, we avoided a cubic equation in [PI] by making two assumptions about $\mathrm{K_d^{inhibitor}}$. First, we assumed that $\mathrm{K_d^{inhibitor}}\ll\ \mathrm{K_d^{ATP}}$, which holds true for any potent competitive inhibitor. This assumption is justified by the observation that even the weaker inhibitor, AzaU, can inhibit 100 μM LuxO at a concentration of <100 μM while competing with a 20-fold higher concentration of ATP. Second, we assumed that $\mathrm{K_d^{inhibitor}}\ll\ \mathrm{P_{tot}}+\mathrm{I_{tot}}$. This assumption is justified by the high concentration of LuxO-C (i.e., $\mathrm{P_{tot}}$ = 100 μM) used in our experiments to ensure that almost all of the enzyme was in the hexameric form.

Armed with an expression for α in terms of the total concentrations of protein, substrate, and inhibitor and the parameter $\mathrm{K}=\mathrm{K_d^{ATP}}/\mathrm{K_d^{inhibitor}}$, we can calculate the probability of having exactly n inhibitors bound to a single oligomer using the binomial distribution. In accord with the results of our analytical ultracentrifugation experiments, we assume six active sites per ring.

$$\rho(n)=\binom{6}{n}\alpha^n(1-\alpha)^{6-n}$$

In this expression, $\binom{6}{n}$ is the number of possible ways of selecting n sites out of 6, and $\alpha^n(1-\alpha)^{6-n}$ is the probability of having n sites occupied while the other 6 − n are empty.

Now let f(n) be the relative inhibition of a single hexamer unit when it has n inhibitors bound. In the simplest case, only the number of bound inhibitors, and not their relative positions, is relevant for enzyme activity. With such a function, albeit unknown, we can link the experimental relative inhibition values to the model using the equation:

$$\text{relative inhibition} = \sum_{k=0}^{6} f(n)\rho(n)$$

The function f relates the number of bound inhibitor molecules n to a degree of inhibition between 0 (no inhibition) and 1 (complete inhibition).

If, as a limiting case, we assume that each bound inhibitor molecule inhibits 1/6th of a LuxO-RC hexamer's enzymatic activity, then:

$$f(n) = \frac{n}{6}$$

and the relative inhibition becomes:

$$\text{relative inhibition} = \sum_{k=0}^{6} \frac{k}{6}\rho(k)$$

At all values of the adjustable parameter K, this model predicts much lower relative inhibition values than we observe.

At the other extreme, we consider the limiting case in which the binding of the first inhibitor molecule to a LuxO-C hexamer yields complete inhibition:

$$f(n) = \begin{cases} 0 & n = 0 \\ 1 & n > 0 \end{cases}$$

In this case, the expression for relative inhibition becomes:

$$relative\ inhibition = \sum_{k=0}^{6} f(k)\rho(k) = \sum_{k=1}^{6} \rho(k) = 1 - \rho(0)$$

Strikingly, we found that we can fit all of our AzaU and CV-133 inhibition data using this model. We did, of course, need to use different values of K for AzaU and CV-133, which inhibit LuxO-C with different potencies. The optimal K values are provided in the text.

We also tested a variety of alternative models. Even relatively minor adjustments, such as setting f(1) = 0.75 and f(n≥2) = 1, prevented us from obtaining the quality of fit achievable with the limiting-case model in which f(1) = 1.

We note that our model avoids the need to consider cooperativity among inhibitor binding sites, as only a single bound inhibitor provides all – or, more realistically, most – of the inhibitory effect. Thus, while we cannot exclude more complex models with additional adjustable

parameters, the parsimony and quality of fit provided by the single-site inhibition model are appealing features.
