# Supplementary material for: Structure, Regulation, and Inhibition of the Quorum-Sensing Signal Integrator LuxO
Source: PLoS Biol. 2016 May 24;14(5):e1002464. doi: 10.1371/journal.pbio.1002464 (PMC4878744; doi:10.1371/journal.pbio.1002464)
Supplement: S1 Table — pET21b-based expression plasmids were generated from genomic DNA using the primers provided here. (PDF) [file pbio.1002464.s007.pdf]

Vibrio species	Domains	Primers	
V. angustum	RCD (1-480)	Forward	ACAAACAACATATGATGCAGCAACGCCAAG
		Reverse	AAGAGACTCGAGTTAATCCATCTCCCTAGCAC
	RC (1-387)	Forward	ACAAACAACATATGATGCAGCAACGCCAAG
		Reverse	AAACTTCTCGAGAATAGGTGGCGGAACC
	CD (141-480)	Forward	AGGCACGCCATATGGGATTTATTGGTAATAG
		Reverse	AAGAGACTCGAGTTAATCCATCTCCCTAGCAC
	C (141-387)	Forward	AGGCACGCCATATGGGATTTATTGGTAATAG
		Reverse	AAACTTCTCGAGAATAGGTGGCGGAACC
V. cholerae	RCD (1-469)	Forward	GCCGACCCATATGCAACATAATCAATCTTTGC
		Reverse	GGCACTCGAGCCGTTCCTTCTCTTTTTC
	RC (1-391)	Forward	GCCGACCCATATGCAACATAATCAATCTTTGC
		Reverse	CAATCTCGAGATTAAGAGGAGGAGGCAGCAT
	CD (148-469)	Forward	CGTAATCCATGGGTAGCAGTCAAACCATGC
		Reverse	GCGTCCTCGAGCGTTCCTTCTCTTTTTCTT
	C (148-391)	Forward	CGTAATCCATGGGTAGCAGTCAAACCATGC
		Reverse	AATTACTCGAGCCAAGAGGAGGAGGCAGCAT
V. vulnificus	RCD (1-466)	Forward	AAAAAAAACATATGATGCAACAAATAACGACA ACG
		Reverse	TTTCATTCGCTCGAGTTATGCTTTC
	RC (1-391)	Forward	AAAAAAAACATATGATGCAACAAATAACGACA ACG
		Reverse	AATAACTCGAGCAAAGGAGGCGGC
	CD (145-466)	Forward	AAGTGATACATATGGGCTTTATCGGCAG
		Reverse	TTTCATTCGCTCGAGTTATGCTTTC
	C (145-391)	Forward	AAGTGATACATATGGGCTTTATCGGCAG
		Reverse	AATAACTCGAGCAAAGGAGGCGGC

	R + linker (1-144)	Forward	AAAAAAAACATATGATGCAACAAATAACGACAACG
		Reverse	TTTCATTCGCTCGAGTTATTGATAATTTTGGCTGCC
	R (1-128)	Forward	AAAAAAAACATATGATGCAACAAATAACGACAACG
		Reverse	TTTCATTCGCTCGAGTTACGCTTTGCGGATAG
V. fischeri	RCD (1-476)	Forward	CCCGCCCGCATATGATGATACAAAAAAAAT
		Reverse	CGCTGTCTACTCGAGTTATCTTTCATTC
	RC (1-389)	Forward	CCCGCCCGCATATGATGATACAAAAAAAAT
		Reverse	ATTGATCTCGAGTAATGGAGGAGGC
	CD (143-476)	Forward	GGGTGGGTCATATGGGATTTATTGG
		Reverse	CGCTGTCTACTCGAGTTATCTTTCATTC
	C (146-393)	Forward	TCTTTCTTCATATGGGTTTTATCGGCAGCAGTC
		Reverse	TAACAACTCGAGTAGCGGTGGCGGTAAC
V. anguillarum	RCD (1-469)	Forward	TCTCTCTCATATGATGCAACCTGACTTTTC
		Reverse	TTTCTTCTCGAGCTATGAGTTCTCTCTACTG
	RC (1-392)	Forward	TCTCTCTCATATGATGCAACCTGACTTTTC
		Reverse	TAACAACTCGAGTAGCGGTGGCGGTAAC
	CD (146-469)	Forward	TCTTTCTTCATATGGGTTTTATCGGCAGCAGTC
		Reverse	TTTCTTCTCGAGCTATGAGTTCTCTCTACTG
	C (146-392)	Forward	TCTTTCTTCATATGGGTTTTATCGGCAGCAGTC
		Reverse	TAACAACTCGAGTAGCGGTGGCGGTAAC
V. parahaemolyticus	RCD (1-467)	Forward	AAAAAAAACATATGATGCAACAAAAAACTGAAGGCC
		Reverse	ACAGTCCTCGAGTTACACCTTC
	RC (1-392)	Forward	AAAAAAAACATATGATGCAACAAAAAACTGAAGGCC
		Reverse	TTATTTCTCGAGCAGCGGCGGTG
	CD (146-467)	Forward	TGGCTAGGCATATGGGTTTTATCGG
		Reverse	ACAGTCCTCGAGTTACACCTTC

	C (146-392)	Forward	TGGCTAGGCATATGGGTTTTATCGG
		Reverse	TTATTTCTCGAGCAGCGGCGGTG
