# Supplementary material for: Structure, Regulation, and Inhibition of the Quorum-Sensing Signal Integrator LuxO
Source: PLoS Biol. 2016 May 24;14(5):e1002464. doi: 10.1371/journal.pbio.1002464 (PMC4878744; doi:10.1371/journal.pbio.1002464)
Supplement: S2 Table — (PDF) [file pbio.1002464.s008.pdf]

Structure	LuxO-C	LuxO-RC
**Data Collection**		
Resolution (Å)	35 – 1.50	35 – 1.60
Outer Shell (Å)	1.53 – 1.50	1.63 – 1.60
Observations	338,644	563,802
Unique Reflections	43,619	72,082
Redundancy	7.8 (7.0)	7.8 (7.3)
Completeness	1.00 (0.99)	1.00 (1.00)
$\langle I/\sigma_I \rangle$	20.9 (4.0)	20.1 (3.3)
R_{merge}	0.057 (0.336)	0.048 (0.651)
R_{meas}	0.061 (0.362)	0.052 (0.700)
R_{pim}	0.021 (0.131)	0.018 (0.257)
$CC_{1/2}$	0.99 (0.97)	0.93 (0.88)
**Refinement**		
Resolution (Å)	25 – 1.5	23 – 1.6
R_{work}	0.162	0.171
R_{free}	0.183	0.196
No. of reflections	43,507	72,021
Completeness	0.998	0.999
No. of atoms	2,273	3,553
No. of waters	221	448
RMS Δbond (Å)	0.010	0.010
RMS Δangle (^{o})	1.14	1.05
RMS B-factor (Å^2)	4.7	4.6
Average B-factor (Å^2)	29.9	29.4
Wilson B-factor (Å^2)	20.8	21.3
Ramachandran Plot		
Favored (%)	98.9	98.2
Outliers (%)	0.0	0.5
PDB code	5EP1	5EP0
