# Supplementary material for: Structure, Regulation, and Inhibition of the Quorum-Sensing Signal Integrator LuxO
Source: PLoS Biol. 2016 May 24;14(5):e1002464. doi: 10.1371/journal.pbio.1002464 (PMC4878744; doi:10.1371/journal.pbio.1002464)
Supplement: S3 Table — (PDF) [file pbio.1002464.s009.pdf]

Structure	LuxO-C:AzaU	LuxO-C:CV-133	LuxO-C:ATP
**Data Collection**			
Resolution (Å)	35 – 1.42	22 – 1.80	35 – 1.50
Outer Shell (Å)	1.50 – 1.42	1.83 – 1.80	1.53 – 1.50
Observations	598,151	132,416	311,193
Unique Reflections	51,077	25,305	40,275
Redundancy	11.7 (8.8)	5.2 (5.1)	7.7 (7.4)
Completeness	1.00 (0.92)	0.99 (1.00)	1.00 (1.00)
$\langle I/\sigma_I \rangle$	31.5 (3.2)	26.5 (4.0)	17.5 (2.3)
R_{merge}	0.058 (0.566)	0.043 (0.546)	0.058 (0.806)
R_{meas}	0.060 (0.601)	0.048 (0.611)	0.062 (0.866)
R_{pim}	0.017 (0.201)	0.021 (0.269)	0.022 (0.315)
$CC_{1/2}$	n/d	n/d	0.98 (0.91)
**Refinement**			
Resolution (Å)	35 – 1.42	21 – 1.80	15 – 1.50
R_{work}	0.160	0.194	0.155
R_{free}	0.184	0.232	0.182
No. of reflections	51,066	24,512	40,145
Completeness	0.996	0.966	0.998
No. of atoms	2,290	2,271	2,311
No. of waters	237	224	237
RMS Δbond (Å)	0.013	0.004	0.016
RMS Δangle (^{o})	1.58	0.65	1.66
RMS B-factor (Å^2)	3.6	4.6	4.4
Average B-factor (Å^2)	25.7	30.8	30.4
Wilson B-factor (Å^2)	17.4	25.0	22.6
Ramachandran Plot			
Favored (%)	98.8	98.1	98.8
Outliers (%)	0.0	0.0	0.0
PDB code	5EP2	5EP3	5EP4
