# Supplementary material for: Structure, Regulation, and Inhibition of the Quorum-Sensing Signal Integrator LuxO
Source: PLoS Biol. 2016 May 24;14(5):e1002464. doi: 10.1371/journal.pbio.1002464 (PMC4878744; doi:10.1371/journal.pbio.1002464)
Supplement: S4 Table — Lowercase letters indicate the altered nucleotide(s). (PDF) [file pbio.1002464.s010.pdf]

| LuxO allele | Primer sequence |
|---|---|
| D61A forward | GCCTGACTTAATCCTGcTGGCTTTACGTTTGCCAGATATGA |
| D61A reverse | TCATATCTGGCAAACGTAAAGCCAgCAGGATTAAGTCAGGC |
| F108A forward | TGCGTCATGGTGCGCAAGACgcTTTGATCAAGCCGTGTGAAG |
| F108A reverse | CTTCACACGGCTTGATCAAAgcGTCTTGCGCACCATGACGCA |
| F108W forward | GCGTCATGGTGCGCAAGACTggTTGATCAAGCCGTGTGAAGC |
| F108W reverse | GCTTCACACGGCTTGATCAAccAGTCTTGCGCACCATGACGC |
| V120E forward | TGAAGCGGACCGACTGCGGGaaACAGTGAATAATGCGATTCG |
| V120E reverse | CGAATCGCATTATTCACTGTttCCCGCAGTCGGTCCGCTTCA |
| G145E forward | AAAACTATCAAGGCTTTATTttTAGCAGTCAAACCATGCAGG |
| G145E reverse | CCTGCATGGTTTGACTGCTAaaAATAAAGCCTTGATAGTTTT |
| G333A forward | GATTGCTTATTCACTGCTTGcCTTTATGTCCAAGGAAGAGG |
| G333A reverse | CCTCTTCCTTGGACATAAAGgCAAGCAGTGAATAAGCAATC |
| G333K forward | GATTGCTTATTCACTGCTTaaaTTTATGTCCAAGGAAGAGGG |
| G333K reverse | CCCTCTTCCTTGGACATAAAtttAAGCAGTGAATAAGCAATC |
